# Erg25 Controls Host-Cholesterol Uptake Mediated by Aus1p-Associated Sterol-Rich Membrane Domains in *Candida glabrata*

**DOI:** 10.3389/fcell.2022.820675

**Published:** 2022-03-24

**Authors:** Michiyo Okamoto, Azusa Takahashi-Nakaguchi, Kengo Tejima, Kaname Sasamoto, Masashi Yamaguchi, Toshihiro Aoyama, Minoru Nagi, Kohichi Tanabe, Yoshitsugu Miyazaki, Hironobu Nakayama, Chihiro Sasakawa, Susumu Kajiwara, Alistair J. P. Brown, Miguel C. Teixeira, Hiroji Chibana

**Affiliations:** ^1^ Medical Mycology Research Center, Chiba University, Chiba, Japan; ^2^ School of Life Science and Technology, Tokyo Institute of Technology, Yokohama, Japan; ^3^ Department of Electronic and Information Engineering, Suzuka National College of Technology, Suzuka, Japan; ^4^ National Institute of Infectious Diseases, Tokyo, Japan; ^5^ Department of Food Science and Human Nutrition, Faculty of Agriculture, Ryukoku University, Otsu, Japan; ^6^ Faculty of Pharmaceutical Sciences, Suzuka University of Medical Science, Suzuka, Japan; ^7^ Nippon Institute for Biological Science, Tokyo, Japan; ^8^ MRC Centre for Medical Mycology, University of Exeter, Exeter, United Kingdom; ^9^ Department of Bioengineering, Instituto Superior Técnico, Universidade de Lisboa, Lisbon, Portugal

**Keywords:** pathogenicity, plasma membrane, C4-sterol methyl oxidase (SMO), virulence factor, opportunistic pathogen, non-albicans, membrane compartment, micro domain

## Abstract

The uptake of cholesterol from the host is closely linked to the proliferation of pathogenic fungi and protozoa during infection. For some pathogenic fungi*,* cholesterol uptake is an important strategy for decreasing susceptibility to antifungals that inhibit ergosterol biosynthesis. In this study, we show that *Candida glabrata ERG25*, which encodes an enzyme that demethylates 4,4-dimethylzymosterol, is required for cholesterol uptake from host serum. Based on the screening of *C. glabrata* conditional knockdown mutants for each gene involved in ergosterol biosynthesis, *ERG25* knockdown was found to decrease lethality of infected mice. *ERG25* knockdown impairs the plasma membrane localization of the sterol importer Aus1p, suggesting that the accumulated 4,4-dimethylzymosterol destabilizes the lipid domain with which Aus1p functionally associates. *ERG25* knockdown further influences the structure of the membrane compartment of Can1p (MCC)/eisosomes (ergosterol-rich lipid domains), but not the localization of the membrane proteins Pma1p and Hxt1p, which localize to sterol-poor domains. In the sterol-rich lipid domain, Aus1p-contining domain was mostly independent of MCC/eisosomes, and the nature of these domains was also different: Ausp1-contining domain was a dynamic network-like domain, whereas the MCC/eisosomes was a static dot-like domain. However, deletion of MCC/eisosomes was observed to influence the localization of Aus1p after Aus1p was transported from the endoplasmic reticulum (ER) through the Golgi apparatus to the plasma membrane. These findings suggest that *ERG25* plays a key role in stabilizing sterol-rich lipid domains, constituting a promising candidate target for antifungal therapy.

## Introduction

Ergosterol is a significant component of the plasma membrane in fungi and protozoa, and its biosynthetic pathway has been successfully used as target in antifungal therapy. However, some pathogenic fungi and protozoa, such as *Candida glabrata*, *Aspergillus fumigatus*, and Trypanosoma brucei, have the ability to scavenge cholesterol from host-serum and utilize it as a surrogate for ergosterol (Coppens and Courtoy, 2000; Bard et al., 2005; Xiong et al., 2005; Nakayama et al., 2007; Nagi et al., 2013). Therefore, there is concern that the uptake of host cholesterol may decrease the susceptibility of these pathogens to antifungal drugs that target ergosterol biosynthesis. Elucidating the molecular mechanisms of cholesterol uptake will facilitate the development of more effective treatments for these fungal and protozoa infections.

Among pathogenic yeast, *C. glabrata* constitutes one of the organisms in which host-cholesterol uptake has been identified. *C. glabrata* also has been the focus of research as an opportunistic pathogen, since this fungus causes severe invasive infections associated to high mortality rates (Kullberg and Arendrup, 2015). *C. glabrata* is evolutionarily much closer to the non-pathogenic yeast *Saccharomyces cerevisiae* than is *Candida albicans*, the other well-characterized and common candida species. Almost 90% of *C. glabrata* genes demonstrate inferred orthology to *S*. *cerevisiae* genes (Lelandais et al., 2008), suggesting a strong conservation of physiology between these two species. *S*. *cerevisiae* can take up external cholesterol, but this process occurs only under anaerobic conditions or in cells with defects in heme biosynthesis, a situation that can mimic anaerobic conditions (Lorenz et al., 1986; Lorenz and Parks, 1991; Shianna et al., 2001; Vik and Rine, 2001). On the other hand, *C. glabrata* can take up cholesterol from serum even under aerobic conditions (Nagi et al., 2013). Therefore, *C. glabrata* appears to be an appropriate tool for investigating the molecular mechanism of host-cholesterol uptake.

*C*. *glabrata* displays intrinsically low susceptibility to azole drugs, like fluconazole, that target ergosterol biosynthesis, specifically inhibiting lanosterol 14-demethylase (Erg11p) activity. The expression of *ERG11,* of the transcription factor encoding gene *PDR1* and of its targets *CDR1*, *CDR2* and *SNQ2*, encoding multidrug transporters, is increased upon treatment with fluconazole (Henry et al., 2000; Sanglard et al., 2009; Vu et al., 2019), resulting in decreased susceptibility to azoles. In addition, the expression of some Drug:H^+^ Antiporters of the Major Facilitator Superfamily, MFS, has also been associated with decreased azole susceptibility (Costa et al., 2014; Cannon and Holmes, 2015). In *C*. *glabrata,* the ATP-binding cassette transporter Aus1p has been shown to mediate cholesterol uptake, its expression being activated by Upc2A*,* a transcriptional activator of ergosterol biosynthesis genes (Nakayama et al., 2007). Interestingly, the expression of *PDR1* and *CDR1* is also dependent on Upc2A (Vu et al., 2019). The expression of *AUS1* is upregulated in the presence of fluconazole and *aus1*∆ cells are highly susceptible to fluconazole even in the presence of serum (Nagi et al., 2013). Furthermore, deletion of *AUS1* leads to reduced proliferation in mice (Nakayama et al., 2007; Nagi et al., 2013) and its expression is upregulated in response to serum or iron-poor environments, as would occur in the bloodstream of hosts (Nagi et al., 2013). Thus, Aus1p-mediated cholesterol uptake may play an important role in fungal infections, especially in bloodstream infections by *C*. *glabrata*. The detergent resistant membrane domains (DRMs) are resistant to extraction with low-temperature nonionic detergents, and sterol and sphingolipid-enriched. DRMs were used to explain protein-lipid interactions (Shogomori and Brown, 2003; Lichtenberg et al., 2005). Recently, DRMs have been used less as an experimental material to reflect the Lipid raft concept in cell membranes (Lingwood and Simons, 2010), however they can be easily tailored to examine the lateral association between the plasma membrane proteins and lipids. In *S. cerevisiae*, Aus1p has been reported to associate with DRMs (Gulati et al., 2015), but in *C. glabrata*, the association of CgAus1p with DRMs remains speculative.

Can1p (arginine permease) and Pma1p (H^+^-ATPase) have been shown to be compartmentalized into distinct types of domains within the plasma membrane of *S*. *cerevisiae*: membrane compartment of Can1p (MCC) and membrane compartment of Pma1p (MCP), respectively. They appear microscopically to have a non-overlapping distribution with distinct patterns; the MCP domains have a network-like pattern, while the MCC domains have a punctate pattern (Malínská et al., 2003). Thus, the distribution of these proteins shows that the plasma membrane is not composed of a uniform arrangement of proteins and lipids, but rather a patchwork of domains with different compositions of proteins and lipids (Spira et al., 2012). The MCC has been suggested to be enriched in ergosterol (Grossmann and Malinsky, 2007), while the MCP has been suggested to be enriched in sphingolipids (van ’t Klooster et al., 2020). The MCC corresponds to specific membrane invaginations that have been termed eisosomes (Strádalová et al., 2009). In pathogenic fungi, MCC/eisosomes appear to be functionally important, given that the deletion of eisosome-associated protein encoding genes leads to defects in cell wall synthesis, in the formation of invasive hyphal filaments, and in virulence in a murine model of *C*. *albicans* infection (Douglas et al., 2012, 2013; Li et al., 2015; Wang et al., 2016).

In this study, we screened a set of knockdown mutants in ergosterol biosynthetic genes (*ERG1*, *ERG7*, *ERG11*, *ERG25*, *ERG26*, and *ERG27*) to identify new players in cholesterol uptake *in vitro* and *in vivo*. Based on the observation that growth defects imposed by *ERG25* or *ERG26* knockdown are not rescued by the presence of serum, new insights into cholesterol uptake in *C*. *glabrata* were obtained. The role of the demethylation of 4,4-dimethylzymosterol by Erg25p in host-cholesterol uptake, mediated by Aus1p-associated membrane domains is scrutinized.

## Materials and Methods

### Strains and Media

Yeast strains used in this study are listed in Table 1. Yeast cells were grown in rich medium (YPD; 2% peptone, 1% yeast extract, 2% glucose) or minimal medium (SD; 0.17% yeast nitrogen base without amino acids and ammonium sulfate, 2% glucose, 5% ammonium sulfate, and appropriate amino acids) at 30 or 37°C. Bovine serum (Sigma-Aldrich, MO, United States) was added to the medium to a final concentration of 10%. Media supplementation with 20 μg/ml doxycycline (Dox) was used to repress gene expression in Tet-off strains.

**TABLE 1:** List of strains used in this study.

Strain	Parent: Modified genotype	References
ACG4	2001HT: *his3 trpl PScHOPZ-tetR-GAL4AD*::*TRP1*	(Nakayama et al., 1998)
HETS202	ACG4: FRT-*YKU80*	(Ueno et al., 2007)
KUE100	*his3 yku80*::*SAT1* flipper	(Ueno et al., 2007)
Tet-ERG1	HETS202: tet97p-*ERG1*::*CgHIS3*	This study
Tet-ERG7	ACG4: tet97p-*ERG7*::*CgHIS3*	This study
Tet-ERG11	ACG4: tet99p-*ERG11*::*CgHIS3*	This study
Tet-ERG25	HETS202: tet99p-*ERG25*::*CgHIS3*	This study
Tet-ERG26	HETS202: tet97p-*ERG26*::*CgHIS3*	This study
Tet-ERG27	ACG4: tet97p-*ERG27*::*CgHIS3*	This study
∆*erg11*	KUE100: *ERG11*::*CgHIS3*	This study
∆erg25	CBS138: *ERG25*::*NAT^R^*	This study
Tet-ERG25 Aus1G	Tet-ERG25: *AUS1*-*GFP ScURA3*	This study
WT_Aus1G	HETS202: *AUS1*-*GFP*::*ScURA3*	This study
WT_Aus1G/Pma1R	HETS202: *AUS1*-*GFP*::*ScURA3*, *PMA1*-*mCherry*::*natNT2*	This study
Tet-ERG25_ Aus1G/Hxt1R	Tet-ERG25: *AUS1*-*GFP*::*ScURA3*, *HXT1*-*mCherry*:: *natNT2*	This study
WT_Aus1G/Pil1R	HETS202: *AUS1*-*GFP*::*ScURA3*, *PIL1*-*mCherry*::*natNT2*	This study
Tet-ERG25_Aus1G/Pil1R	Tet-ERG25: *AUS1*-*GFP*:*ScURA3*, *PIL1*-*mCherry*::*natNT2*	This study
Tet-ERG25 *pil1*∆_Aus1G	Tet-ERG25: ∆*C*::*NAT* ^*R*^ *AUS1-GFP*::*ScURA3*	This study
Tet-ERG26_ Aus1G/Hxt1R	Tet-ERG26: *AUS1*-*GFP*::*ScURA3*, *HXT1*-*mCherry*:: *natNT2*	This study

### Strain Construction

To construct Tet-off strains, *ERG11*, *ERG25* or *PIL1* deletion strain, each DNA cassette was amplified by polymerase chain reaction (PCR) using the primers listed in Supplementary Table S1 and plasmid pTK916-97t (Ueno et al., 2010), pTK916-99t (Niimi et al., 2012), pCgHIS906 containing Cg*HIS3,* or pBM16.1 containing *NAT1* gene (Zordan et al., 2013) as a template. The resulting products were transformed into HETS202 cells, KUE100, or CBS138. Bovine serum was added to the selection medium for the construction of ∆*erg11* cells to maintain the growth of cells, and bovine serum and fluconazole were added for the construction of ∆*erg25* cells. Transformed cells were screened by colony PCR to verify that the tetracycline-dependent down-regulatable promoter had been inserted upstream of each target gene in the parent strain. Transformation and colony PCR methods were described in our previous report (Ueno et al., 2011). Strains expressing Aus1p-GFP, Hxt1p-mCherry, or Pil1p-mCherry were constructed using a PCR-based method with an integrative cassette. The cassette for tagging Aus1p with GFP was amplified using primers pAUS1F′ and pAUS1R’ (Supplementary Table S1) and genomic DNA derived from the UTH*aus1*∆/*AUS1*-*GFP* strain (Nagi et al., 2013) as template. *GFP-ScURA3* was inserted into the downstream end of *AUS1 via* homologous recombination. To tag Hxt1p with mCherry, Fragment 1 (encoding mCherry-natNT2) was amplified using primers pFA6aF and pFA6aR and plasmid pFA6a-mCherry-natNT2 (Okamoto et al., 2012) as template. Fragment 2, containing the downstream end of the *HXT1* open reading frame (ORF), was amplified using primers HXT1F1 and HXT1R and *C*. *glabrata* genomic DNA of HETS202 as template. Fragment 3, containing sequences downstream of the *HXT1* ORF, was amplified using primers HXT1F2 and HXT1R2 and *C*. *glabrata* genomic DNA as template. Fusion PCR was carried out using primers HXT1F1 and HXT1R2 and Fragments 1, 2, and 3 as templates, and then sequences encoding mCherry-natNT2 were inserted into the downstream end of the *HXT1* ORF by homologous recombination. The strains expressing Pil1p-mCherry or Pma1p-mCherry were constructed using the aforementioned cassette, derived from each of following primers (pFA6aF, pFA6aR, PIL1F1, PIL1R1, PIL1F2, and PIL1R2) or (pFA6aF, pFA6aR, PMA1F1, PMA1R1, PMA1F2, and PMA1R2), and using the same PCR method as employed for the mCherry-tagging of Hxt1p. Insertion of these cassettes into transformed cells was verified by colony PCR.

### qRT-PCR

Cells were grown in minimal medium at 37°C overnight. This overnight pre-culture was used to inoculate a fresh culture at a density of 1  ×  10^7^ cells/ml in minimal medium in the presence or absence of 20 μg/ml Dox; the resulting culture was incubated at 37°C for 4 h with shaking. Cells then were collected by centrifugation and washed twice with sterile distilled water at 4°C. Total RNA was extracted using the RNeasy Mini extraction kit (Qiagen, Hilden, Germany). cDNA was synthesized from the total RNA using ReverTra Ace and random primers (Toyobo, Osaka, Japan). The amount of RNA for each gene was determined by quantitative real-time PCR (qRT-PCR) on a LightCycler^®^ 96 System (Roche Diagnostics, Mannheim, Germany) with SYBR Green detection using the Thunderbird SYBR qPCR mix (Toyobo). Transcript levels were normalized to that of *TEF1*, a housekeeping gene that encodes elongation factor 1. PCR conditions were as follows: pre-denaturation at 95°C for 1 min, followed by 40 cycles of denaturation at 95°C for 15 s and annealing/extension at 60°C for 1 min.

### Assay of NBD-Cholesterol Uptake

Cells were grown to early exponential phase in minimal medium and then subcultured for 17 h in minimal medium containing 10% (v/v) bovine serum, 0.1% (w/v) Tween 80, and 5 μg/ml NBD-cholesterol (25-[N-[(7-nitro-2-1,3-benzoxadiazol-4-yl)methyl]amino]-27-norcholesterol; Avanti Polar Lipids, AL, United States) with or without Dox (20 μg/ml). Following culturing, the cells were washed twice with ice-cold phosphate-buffered saline (PBS) containing 0.5% (w/v) Nonidet P-40 and then once with PBS; the resulting pellet was resuspended in PBS. The cells were observed using a fluorescence microscope equipped with an NIBA filter (BX53; Olympus, Tokyo, Japan), and fluorescence intensity was quantified by flow cytometry (FACSVerse; Becton Dickinson, NJ, United States). Cells (1 ml volume of cells suspended in PBS) were pre-stained by the addition of propidium iodide (1 µl of a 1-mg/ml solution) to exclude dead cells from the analysis. Flow cytometry was performed as described previously (Marek et al., 2014). Cells cultured without NBD-cholesterol were analyzed as a control.

### DRM Isolation and Immunoblotting

Cells were grown to logarithmic phase at 37°C in minimal medium containing 10% serum and 0.05% Tween 80 and incubated for 5.5 h after addition of 20 μg/ml Dox. Upon reaching an optical density at 600 nm (OD_600_) of 20, the cells were treated with 10 mM NaN_3_ and resuspended in TNE buffer (25 mM Tris-HCl [pH 7.5], 150 mM NaCl, and 5 mM EDTA) containing complete protease inhibitor cocktail (Sigma-Aldrich) and 1 mM PMSF. Cells were disrupted with glass beads using a Multi-beads Shocker (Yasui Kikai, Osaka, Japan). Debris and unbroken cells were removed by centrifugation for 5 min at 500× *g*. After incubation with 1% Triton X-100 (Sigma-Aldrich) for 30 min on ice, 840 µl of Optiprep solution (Alere Technologies AS, Oslo, Norway) was added to the lysate (420 µl) for a final Optiprep concentration of 40%, and the lysate was placed in a centrifuge tube. The sample was sequentially overlaid with 2.0 ml of 30% Optiprep (in TNE plus 0.1% Triton X-100) and 330 µl of TNE buffer containing 0.1% Triton X-100. The tube then was subjected to Optiprep density gradient flotation by centrifugation for 3.5 h at 35,000 rpm (168,000 g) in a P40ST rotor with a 4S13 adapter (Eppendorf Himac Technologies, Ibaraki, Japan) at 4°C. After centrifugation, 7 fractions of equal volume were collected starting from the top. Proteins in each fraction were precipitated with trichloroacetic acid (TCA) and resuspended in sample buffer. After incubation at 37°C for 10 min, the samples were resolved by 7.5% SDS-PAGE, transferred to a PVDF membrane (Immobilon-P, Merck Millipore, MA, United States), and subjected to western blotting. Western blotting was carried out using JL-8 anti-GFP monoclonal antibody (Clontech Laboratories, CA, United States) or anti-Pma1p polyclonal antibody (sc-33735, Santa Cruz Biotechnology, TX, United States).

### Fluorescence Microscopy Analyses

The fluorescence microscopic images were observed with a BZ-9000 (Keyence, Osaka, Japan) equipped with a 100x oil-immersion objective lens or an Axio Observer Z.1 (Carl Zeiss, Jena, Germany) equipped with a 100x oil-immersion objective and a CMOS camera (Touptec photonics, Hangzhou, China). Basically, images were acquired with 1 s exposure. Confocal microscopy images were detected with a Stellaris 5 (Leica Microsystems, Wetzlar, Germany) equipped with a 100x objective lens and processed by lightning deconvolution on LAS X software. FM4-64 (FUJIFILM Wako Pure Chemical corporation, Osaka, Japan) was used to stain the vacuolar membrane. The cells expressing Aus1p-GFP were grown exponentially at 37°C in serum-containing SD medium and further incubated with or without Dox for 1 h. The FM4-64 stock solution (1.6 µM in DMSO) was added at a final concentration of 10 µM. After 30 min of incubation, cells were washed and further incubated with or without Dox for 1.5 h. To examine sterol localization, cells co-expressing Aus1p-GFP and Pma1p-mCherry were incubated with 5 μg/ml filipin (Polysciences, PA, United States) for 5 min at 30°C. After three rinses with PBS, the stained cells were observed by confocal fluorescence microscopy using excitation wavelengths of 405 nm for filipin, 488 nm for GFP, and 568 nm for mCherry. The brightness and contrast of images were adjusted with Fiji/ImageJ software (Schindelin et al., 2012). The EzColocalization plug-in was used for colocalization analysis and the assessment of Pearson’s correlation coefficient.

### Sterol Analysis

Cells were incubated under the conditions described above for the isolation of DRMs. After quantification of total protein using the Bradford method, DRMs were isolated by Optiprep density gradient flotation. Sterols were analyzed using GC. After cultivation, cells were harvested and freeze-dried to determine dry weight, and dried cells were resuspended in 4 ml of methanol. Following addition of an internal standard, 5-α-cholestane was added at 2 μg/g cell dry weight, and the cells were homogenized on ice for 10 min using a Hom-100 subsonic homogenizer (AGC Techno Glass, Shizuoka, Japan). The cell homogenate was transferred to a new tube, and 20% (w/w) solid KOH was added. The mixture then was vortexed until the KOH was completely dissolved. Lipids in DRMs were saponified at 85°C for 2 h. After the saponified mixture was cooled to room temperature, 4 ml of hexane and 1 ml of distilled water were added to extract alkali-stable lipids. After washing with 4 ml of distilled water, the extract was dried and then trimethylsilylated in pyridine at 70°C for 1 h using *N*,*O*-bis (trimethylsilyl) trifluoroacetamide (Tokyo Chemical Industry, Tokyo, Japan). After cooling to room temperature, the trimethylsilylated sterols were analyzed by GC (GC-18A, Shimadzu, Kyoto, Japan) using a 0.25 mm × 30 m Rtx-35MS column (Restek Corp., PA, United States) under the following conditions: the initial column temperature of 300°C was maintained for 1 min, increased to 310°C at a rate of 10°C/min, and then maintained at 310°C for an additional 10 min. Trimethylsilylated sterols were detected by reference to pure substances; otherwise, sterols were identified by Shimadzu Corporation using GC-MS analysis.

### Electron Microscopy

Tet-*ERG25* cells were pre-grown to exponential phase in minimal medium, and then inoculated to minimal medium containing serum with or without Dox. After 17 h, cells were collected by brief centrifugation and snap-frozen with melting propane in liquid nitrogen. Samples were freeze-substituted in OsO_4_-acetone at −80°C for 4 days and embedded in epoxy resin. Ultrathin sections were cut to a thickness of 70 nm, stained with uranyl acetate and lead citrate, covered with Super support film (Nisshin EM, Tokyo, Japan), and observed using a JEM-1400 electron microscope (JEOL, Tokyo, Japan). The surface density of furrow-like invaginations was calculated from electron micrographs using the Fiji/ImageJ software.

### Animal Infections

Male mice (6 weeks old, 19–24 g each, BALB/c; purchased from Oriental Yeast, Japan) were immunosuppressed by intraperitoneal injection of cyclophosphamide in saline (at 150 mg/kg body weight) 5 days before and 3 days before the infection, and every 3 days post-infection (dpi) until 27 dpi. Each group consisted of 14 mice. Mice were provided 5% (w/v) sucrose solution with or without Dox (10 mg/ml) as drinking water from 4 days prior to the injection until the end of the in-life interval. In the Dox-treated group, mice were injected intraperitoneally with Dox in PBS at a dose of 300 mg/kg body weight every 3 dpi. Mice were infected with 2 × 10^7^ *C. glabrata* yeast cells in 200 µl of saline via tail vein injection. The mice experiments were performed strictly according to the guidelines of the Animal Care and Use Committee of Chiba University, Japan, which follows the NIH “Guide for the Care and Use of Laboratory Animals”.

## Results

### *ERG25* is Required for *Candida glabrata* Serum-Dependent Growth *In Vitro* and for Lethality in Infected Mice

**FIGURE 1:**
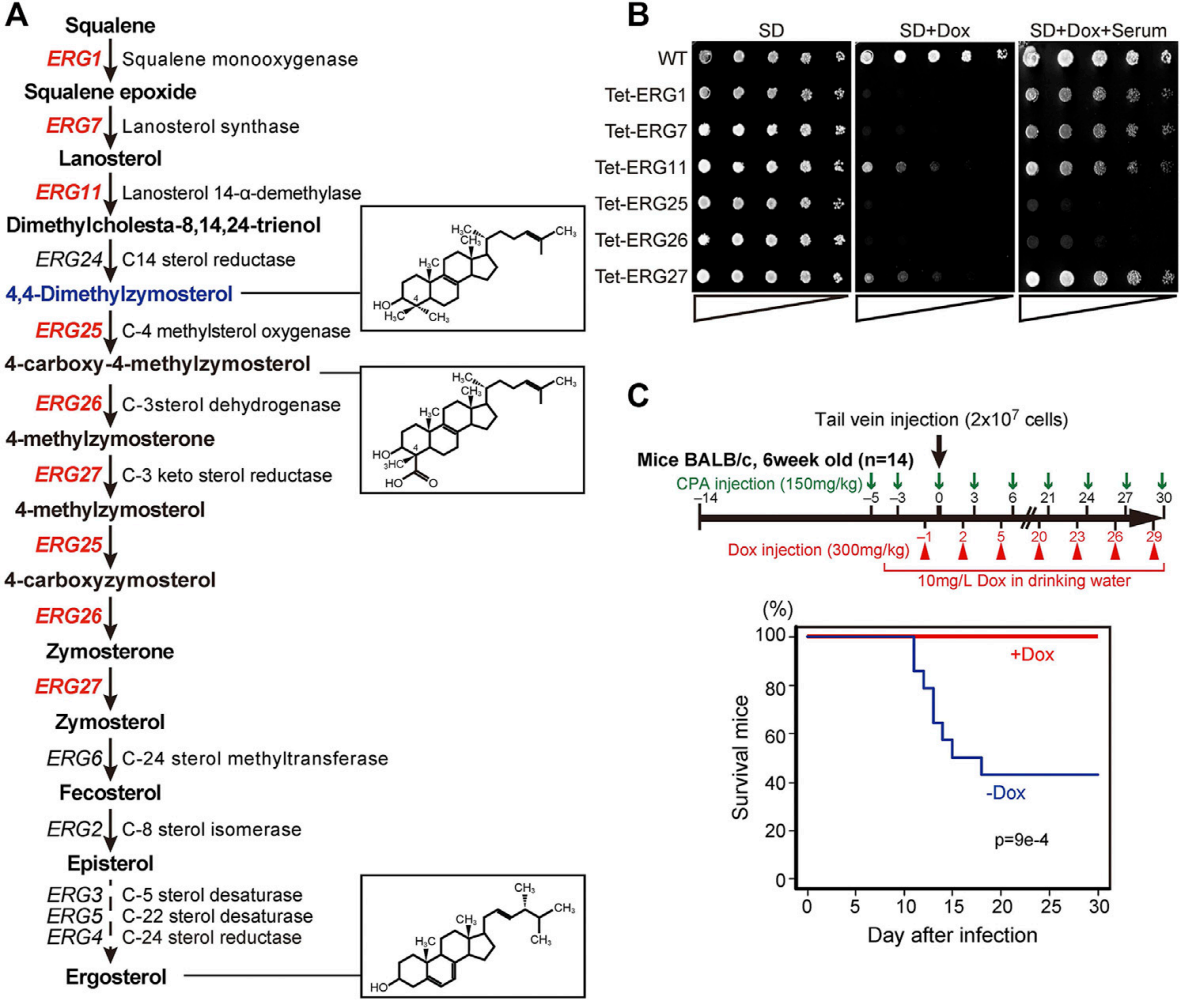
ERG genes and the effects of knockdown. **(A)** Biosynthetic pathway from squalene to ergosterol, including genes, enzymes, and metabolic intermediates. Twelve genes encode enzymes of the ergosterol biosynthetic pathway in *S. cerevisiae*; the six genes in red are essential for growth in *S. cerevisiae*, in the absence of exogenous ergosterol. The chemical structures of ergosterol and 4, 4-dimethylzymosterol are indicated in the boxes to the right. **(B)** Effect of serum supplementation in cells subjected to gene repression. Wild-type (HETS202) and Tet-ERG cells in which the indicated *ERG* genes are knocked down by Dox diluted to OD_600_ (an optical density at 600 nm) of 0.5 in water. The diluted cells were spotted in 4-fold serial dilutions as indicated by triangles on agar plates of minimal medium (SD), SD containing 20 μg/ml Dox (SD + Dox), or SD containing 20 μg/ml Dox and 10% serum (SD + Dox + Serum), and incubated for 24 h at 37°C. The experiment was conducted three times. **(C)** Survival rate of mice after infection with Tet-ERG25 cells. As shown in the flowchart (upper panel), mice were dosed with 150 mg/kg body weight of cyclophosphamide (CPA) 3 and 5 days before the infection, and every 3 days after infection until the end of the experiment. The mice also were injected intraperitoneally with Dox in PBS (300 mg/kg body weight) every 3 days starting 1 day before the infection and also provided with drinking water containing Dox (10 mg/L) from starting from 4 days before infection. Log-phase Tet-ERG25 cells (2 × 10^7^ yeast cells/animal) were administered to mice by tail vein injection. Red and blue lines indicate the survival rates of mice for the Dox-treated and Dox-untreated groups, respectively (*n* = 14 each). Vertical and horizontal axes indicate percentage of mice still alive and days after infection, respectively. *p* values were calculated using the log-rank (Mantel-Cox) test.

Among the ergosterol biosynthetic (*ERG*) genes, encoding enzymes for the conversion of squalene to ergosterol, six are essential for the growth in *Saccharomyces cerevisiae* in the absence of exogenous ergosterol (Figure 1A) (Giaever et al., 2002). The orthologous genes in *C. glabrata* include: *CgERG1* (CAGL0D05940g), *CgERG7* (CAGL0J10824g), *CgERG11* (CAGL0E04334g), *CgERG25* (CAGL0K04477g), *CgERG26* (CAGL0G00594g), and *CgERG27* (CAGL0G00594g). The deletion of each ERG gene results in the accumulation of individual sterol intermediates that are structurally distinct from ergosterol. To investigate whether these various intermediates may cause differences in the effects of cholesterol uptake on growth defects, we constructed conditional knockdown mutants (Tet-ERG) by replacing each promoter with a tetracycline-repressible (Tet-off) promoter (Supplementary Figure S1A). This Tet-off system is a useful tool for functional analysis of essential genes, since gene expression can be knocked down by the addition of tetracycline both *in vitro* and *in vivo* (Nakayama et al., 1998; Nakayama et al., 2000). We then checked the resulting Tet-ERG1, Tet-ERG7, Tet-ERG11, Tet-ERG25, Tet-ERG26, and Tet-ERG27 strains for their ability to grow in serum-supplemented medium. In each strain, the transcription of the corresponding gene was markedly knocked down in the presence of doxycycline (Dox) (Supplementary Figure S1B). Although Tet-ERG11 and Tet-ERG27 cells grew slightly in minimal (SD) medium containing Dox, these Tet-off strains exhibited a clear growth defect compared to the wild type cells (Figure 1B). Supplementation with serum permitted the Tet-ERG1, Tet-ERG7, Tet-ERG11, and Tet-ERG27 cells to grow in the presence of Dox. In contrast, Tet-ERG25 and Tet*-*ERG26 cells grown in the presence of Dox were not rescued by the addition of serum. Furthermore, to confirm whether the effect of knockdown by the Tet-off system on growth strictly reflects the effect of gene disruption, we performed the deletion of *ERG11* and *ERG25.* Similar to the knockdown of *ERG11*, ∆*erg11* cells displayed growth defect in minimal medium, and its growth defect was recovered by the addition of serum (Supplementary Figure S2). On the other hand, ∆*erg25* cells could grow in minimal medium containing serum when Erg11p, which is an upstream enzyme of Erg25p, was inhibited by fluconazole, but not in serum-free medium. *ERG25* encodes C-4 methyl sterol oxidase and *ERG26* encodes C-3 sterol dehydrogenase; together, the two enzymes catalyze a sequence of reactions that convert 4,4-dimethylzymosterol to zymosterone (Figure 1A). To clarify why cholesterol uptake did not rescue the growth defect caused by the inhibition of these demethylation steps, further analysis was performed using Tet-ERG25 cells. We investigated the requirement of Erg25p in blood-stream infection by examining the survival rate of mice administered Tet-ERG25 cells by tail vein injection. Because *Candida* causes an opportunistic infection, the infected host mice were immunosuppressed with cyclophosphamide prior to infection. The mice were infected with Tet-ERG25 cells and fed Dox to repress *ERG25* expression, as described in the Materials and Methods. In the group of mice not receiving Dox, survival decreased rapidly after the 11th day post-infection (dpi). In contrast, all mice receiving Dox survived until, at least, the 30th dpi (Figure 1C). The apparent difference in survival rates between the two groups was statistically significant (*p* < 0.001), revealing that *ERG25* knockdown reduces the lethality of mice infected with *C*. *glabrata*.

### *ERG25* Knockdown Leads to Decreased Cholesterol Uptake and Mis-Targeting of the Cholesterol Transporter Aus1p

**FIGURE 2:**
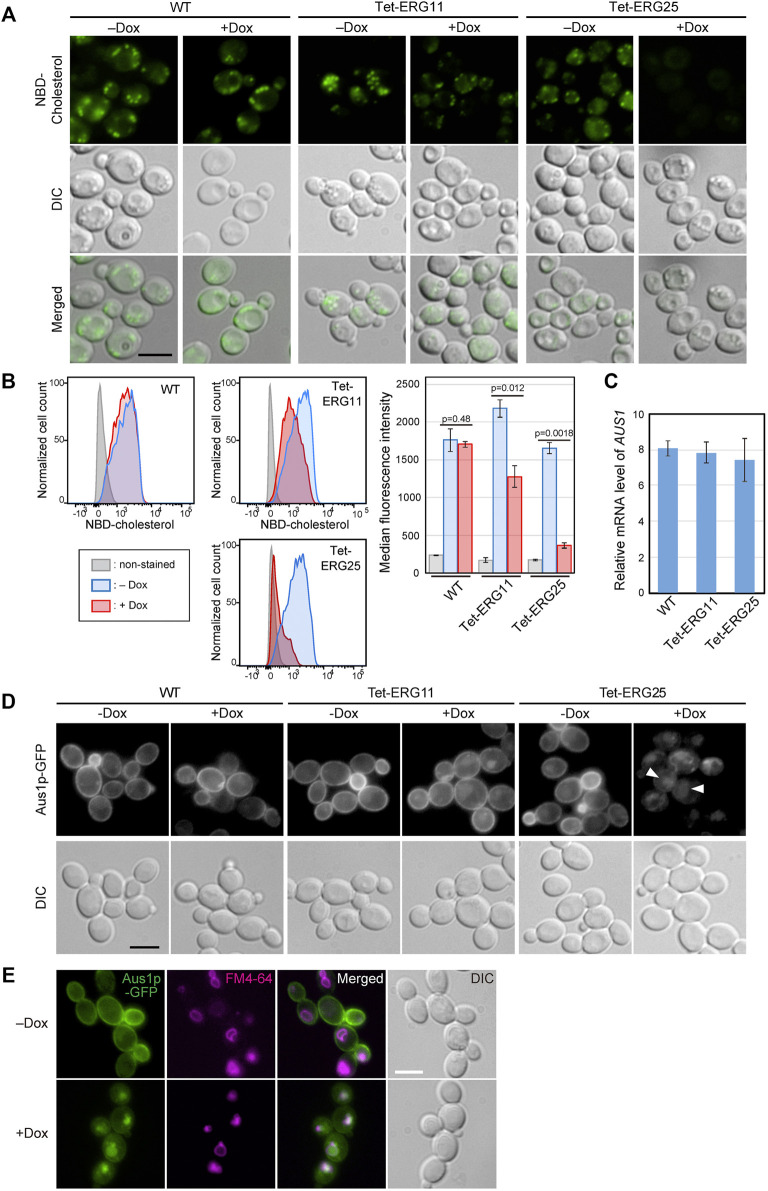
*ERG25* knockdown causes defects in cholesterol uptake and the localization of Aus1p. **(A)** Uptake of fluorescent cholesterol analogues, NBD-cholesterol. Wild-type (HETS202), Tet-ERG11 and Tet-ERG25 cells were incubated with NBD-cholesterol in the presence (+Dox) or absence (–Dox) of Dox for 17 h in minimal medium containing 10% (v/v) serum. After washing, the fluorescence of NBD-cholesterol taken up into each cell was observed under a fluorescence microscope. **(B)** Wild-type, Tet-ERG11 and Tet-ERG25 cells were incubated as described in **(A)**, and then treated with propidium iodide (PI). Cells not stained with PI were defined as living cells, and the fluorescent intensity of NBD-cholesterol taken up into the cells was analyzed by flow cytometry. Cells incubated without NBD-cholesterol served as unstained controls. Cell count was normalized to the peak height at its mode of the distribution by FlowJo software. The maximum value of each peak was converted as 100%. Median fluorescence intensity was quantified from the result of each flow cytometry and represented as a graph. Values are presented as mean ± SD of three independent experiments. **(C)** *AUS1* transcript levels in wild-type (WT; KUE100), Tet-ERG11 and Tet-ERG25 cells. Cells were incubated at 37°C for 4 h in minimal medium with 20 μg/ml Dox in the presence of 10% bovine serum. *AUS1* mRNA was quantified by qRT-PCR, and the data were normalized against the corresponding levels of a housekeeping transcript (*TEF1*). Values are presented as mean ± standard deviation (SD) of three independent experiments. **(D)** Effect of *ERG25* knockdown on the localization of Aus1p. Wild-type, Tet-ERG11 or Tet-ERG25 cells that express Aus1p-GFP (WT_Aus1G, Tet-ERG11_Aus1G or Tet-ERG25_Aus1G) were incubated in minimal medium containing 10% (v/v) serum at 37°C with or without 20 μg/ml Dox. After 3.5 h, fluorescence associated with Aus1-GFP was observed using a microscope. DIC, Differential Interference Contrast. **(E)** *ERG25* knockdown causes the mislocalization of Aus1p to the vacuole. Tet-ERG25 cells that express Aus1p-GFP (Tet-ERG25_Aus1G) were incubated in minimal medium containing 10% (v/v) serum at 37°C with or without 20 μg/ml Dox for 3 h. Vacuole membrane was stained with FM4-64. All scale bars represent 5 µm.

To examine whether cholesterol uptake was functional in *ERG25* knockdown cells, we assessed the uptake of NBD-cholesterol, which is a fluorescently tagged analog of cholesterol, by fluorescence microscopy (Figure 2A). In wild-type and Tet-ERG11 cells grown in the absence of Dox, NBD-cholesterol is detected as clear intracellular punctate structures. Previously, these punctate structures have been described as lipid particles or droplets (Marek et al., 2014).The addition of Dox to wild-type cells did not affected the distribution of NBD-fluorescence. In Tet-ERG11 cells, although the NBD fluorescence was weaker, punctate signals were also observed under Dox conditions (i.e., conditions under which growth is maintained by the addition of serum.). On the other hand, in Tet-ERG25 and Tet-ERG26 cells, NBD-stained punctate structures no longer were observed in the presence of Dox and serum (Figure 2A; Supplementary Figure S3A). Additionally, we quantified NBD-cholesterol import in the cells using flow cytometry (Figure 2B). We pre-stained the cells with propidium iodide (PI), and excluded PI-staining cells from the analysis as dead cells. In wild-type cells, the fluorescence intensity of NBD was high regardless of the presence or absence of Dox. In Tet-ERG11 cells, the fluorescence intensity of NBD in the presence of Dox was lower than that in its absence, but higher than that of the non-stained cells. In Tet-ERG25 cells, the fluorescence intensity in the presence of Dox was clearly lower than that in its absence and was comparable to that of the non-stained cells. These results suggest that *ERG11* knockdown cells can partially take up NBD-cholesterol, while *ERG25* knockdown cells cannot take up NBD-cholesterol at all.

In *C. glabrata*, host-cholesterol uptake is mediated by the ATP-binding cassette transporter Aus1p, the expression of which is upregulated upon addition of serum (Nagi et al., 2013). To determine why *ERG25* knockdown cells are unable to take up cholesterol from serum, we focused on the expression of the *AUS1* gene and localization of Aus1p. We performed quantitative RT-PCR to examine the effect of *ERG25* knockdown on *AUS1* expression in the presence of serum (Figure 2C). The level of *AUS1* mRNA in Tet-ERG25 cells treated with Dox was comparable to those of Dox-treated wild-type and Tet-ERG11 cells. This result suggests that the transcription of *AUS1* is not affected by *ERG25* knockdown.

Next, we investigated the effects of *ERG25* knockdown on Aus1p localization in a Tet-ERG25 strain using an Aus1p-Green Fluorescent Protein (GFP) fusion expressed from the endogenous *AUS1* locus. Since the ∆*aus1* strain is sensitive to fluconazole even in medium containing serum, the fact that the wild-type strain with GFP tagging on *AUS1* is not sensitive to fluconazole in medium containing serum suggests that Aus1p-GFP is functional. In the absence of Dox, the fluorescence of Aus1-GFP was detected on the cell surface in Tet-ERG11 and Tet-ERG25 cells (Figure 2D). In the presence of Dox, Aus1p-GFP localization to the plasma membrane was partially retained in Tet-ERG11 cells. In contrast, in Tet-ERG25 cells, the fluorescence of Aus1p-GFP disappeared from the cell surface; instead, atypical fluorescent clumps were observed (Figure 2D, white arrowhead). The fluorescence intensity profile also indicated that the fluorescence peak indicating localization to the cell membrane disappeared in the presence of Dox in Tet-ERG25 (Figure 2D, black arrowhead). The localization of Aus1p-GFP in Dox treated Tet-ERG25 cells appears to be vacuolar, its signal being surrounded by FM4-64, which stains vacuolar membranes (Figure 2E). These observations suggested that the inability of *ERG25* knockdown cells to uptake exogenous cholesterol is due to mislocalization of Aus1p, which may be unable to sort properly into the plasma membrane.

### *ERG25* Knockdown Leads to Loss of Aus1p-DRMs Association and Altered DRMs Sterol Composition

**FIGURE 3:**
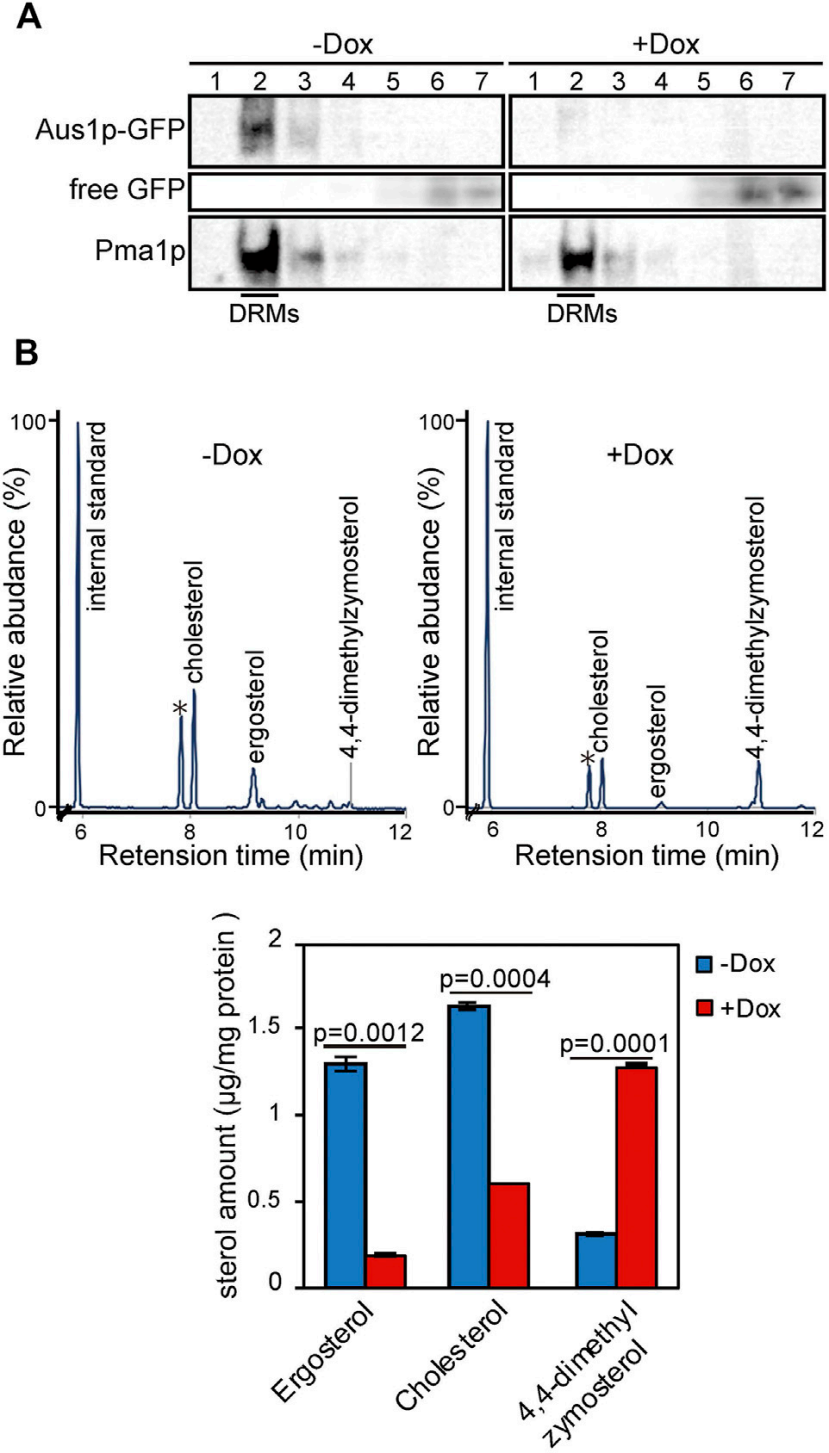
*ERG25* knockdown causes the dissociation of Aus1p from detergent-resistant membranes (DRMs) and a component change of DRMs. **(A)** Biochemical analysis of the effect of *ERG25* knockdown on the association of Aus1p with DRMs. Tet-ERG25 cells that express Aus1p-GFP (Tet-ERG25_Aus1G) were grown to exponential phase at 37°C in minimal medium containing 10% (v/v) bovine serum and then incubated with or without 20 μg/ml Dox for 5.5 h. The cells were disrupted with glass beads, extracted with 1% Triton X-100, and subjected to Optiprep density gradient centrifugation. Seven fractions were collected and analyzed by western blotting with antibodies against GFP and Pma1p. Pma1p was used as a marker for DRM-associated proteins. Raw data was displayed in Supplementary Figure S4. **(B)** Sterol analysis of DRMs in Tet-*ERG25* cells. The DRM fraction was analyzed by gas chromatography. Before the centrifugation, the quantity of total protein was determined by protein assay. 5-α-Cholestane was used as an internal standard. Individual sterols are quantified and indicated in a lower panel. Individual sterols were identified by comparison to standards or GC-MS. Values are presented as mean ± SD of three independent experiments. ∗; Triton-100.

In *S*. *cerevisiae*, plasma membrane proteins have been suggested to associate with DRMs, which are enriched in ergosterol and sphingolipids. DRMs are proposed to be involved in the trafficking of proteins to the plasma membrane. Therefore, we evaluated whether the inability of Aus1p to localize to the plasma membrane in *ERG25* knockdown cells reflected the inability of Aus1p to associate with DRMs. Specifically, we isolated DRMs from Tet-ERG25 cells by the classical method, wherein a cell lysate is treated with Triton X-100 at 4°C and then fractionated by Optiprep density gradient centrifugation. Each fraction was analyzed by immunoblotting with antibodies against GFP to detect Aus1p-GFP or against Pma1p, a representative DRM-associated protein in *S. cerevisiae* (Gulati et al., 2015). When DRMs were isolated from cells grown in the absence of Dox, Pma1p was detected in Fraction 2, indicating that Fraction 2 was enriched in DRMs (Figure 3A). Aus1p-GFP also was detected in Fraction 2, similar to Pma1p. When DRMs were isolated from cells grown in the presence of Dox, Aus1p-GFP was no longer seen in Fraction 2. Instead, we observed an increase in low-molecular-weight proteins, presumably corresponding to free GFP, in the detergent-soluble fraction (Fractions 5-7) compared to cells grown in the absence of Dox. Because GFP tends to be resistant to vacuolar proteases (Conibear and Stevens, 2002), we speculated that the free GFP was derived from vacuolar degradation of Aus1p-GFP. This hypothesis is supported by the localization of the GFP fluorescence to the vacuole in *ERG25* knockdown cells (Figure 2E). In contrast, bands corresponding to Pma1p still were present in Fraction 2. Together, these results suggested that Aus1p associates with DRMs in *C. glabrata*, and that this association is disrupted in the *ERG25* knockdown cells.

To clarify why the association of Aus1p with DRMs is disrupted in the *ERG25* knockdown cells, we focused on 4,4-dimethylzymosterol, the precursor that is expected to accumulate in the *ERG25* knockdown cells. The formation of DRMs depends on specific interactions between sterols and sphingolipids (Klose et al., 2010) and a structural change of sterols affect the association of the plasma membrane with DRMs (Eisenkolb et al., 2002; Umebayashi and Nakano, 2003). We hypothesized that in cells knocked down for *ERG25* expression, the sterol in DRMs would change from ergosterol and cholesterol to 4,4-dimethylzymosterol, thereby affecting the association of Aus1p with DRMs. To test this hypothesis, we performed quantitative gas chromatography analysis (GC) of DRMs derived from Tet-ERG25 cells to investigate whether 4,4-dimethylzymosterol is contained primarily in the DRM fractions (Figure 3B). In the DRMs derived from Tet-ERG25 cells cultured with serum in the absence of Dox, the main sterol components were ergosterol and cholesterol, while 4,4-dimethylzymosterol was a minor sterol. In contrast, in DRMs derived from Tet-ERG25 cells cultured in the presence of Dox, the main sterol component was 4,4-dimethylzymosterol instead of ergosterol and cholesterol. Given that *ERG25* knockdown resulted in replacement of the ergosterol and cholesterol with 4,4-dimethylzymosterol as the DRM-forming sterol, it suggests that 4,4-dimethylzymosterol influences the association of Aus1p with DRMs.

### *ERG25* Knockdown Has Little Effect on the Plasma Membrane Localization of the DRM-Associated Proteins Pma1p and Hxt1p

**FIGURE 4:**
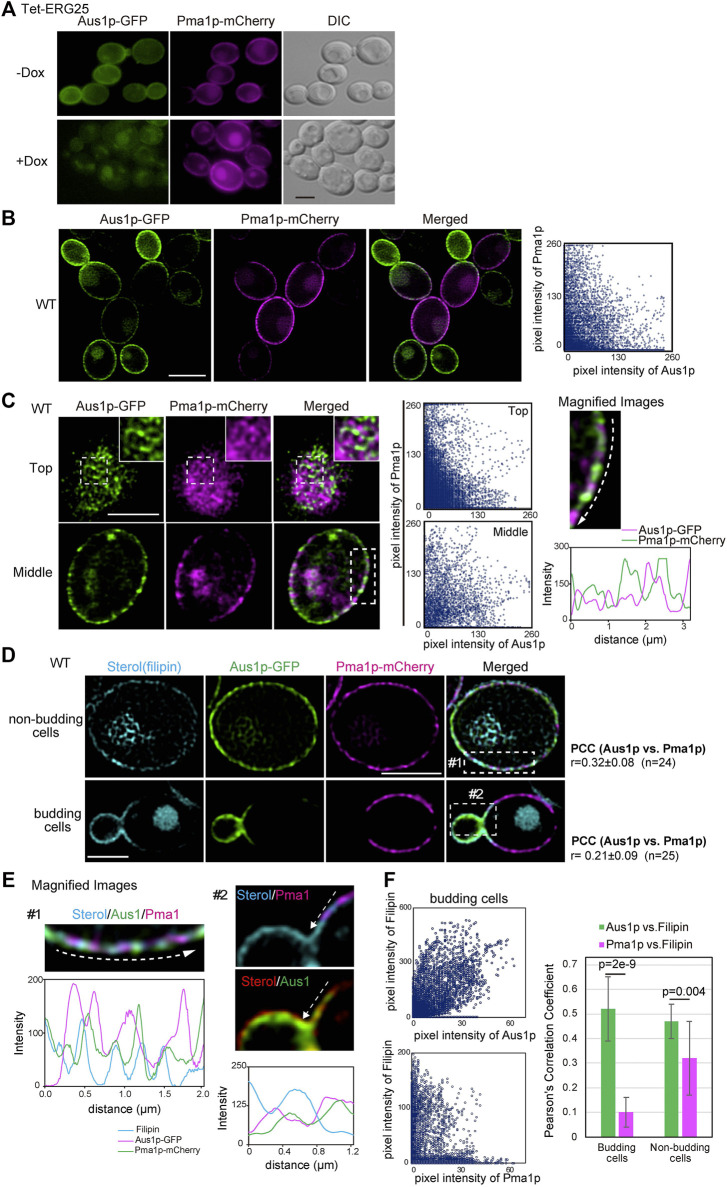
*ERG25* knockdown has no effect on the localization of Pma1p. **(A)** Effect of *ERG25* knockdown on the localization of Pma1p. Tet-ERG25 cells that expresses Aus1p-GFP and Pma1p-mCherry (Tet-ERG25_Aus1G/Pma1R) were grown to exponential phase at 30°C in minimal medium containing 10% (v/v) bovine serum and observed using fluorescence microscopy after incubation with or without 20 μg/ml Dox for 3.5 h. **(B)** Distribution of Aus1p and Pma1p on the plasma membrane. Wild-type cells co-expressing Aus1p-GFP and Pma1p-mCherry (WT_Aus1G/Pma1R cells) were grown to exponential phase at 30°C in minimal medium containing 10% (v/v) bovine serum. Scatterplots of green and magenta pixel intensities of Aus1p-GFP and Pma1p-mCherry were performed using the Fiji/ImageJ software (right panel). **(C)** WT_Aus1G/Pma1R cells were observed at the cell surface (Top) and transverse region (Middle) using a high-resolution confocal fluorescence microscope in real time. Each area enclosed by the dashed lines also is provided as a magnified image. In the magnified image of the transverse region, intensity profiling of GFP (green) and mCherry (magenta) on the plasma membrane was carried out in the direction shown by the arrow. Scatterplots of green and magenta pixel intensities in each panel were indicated. **(D)** Comparison of distribution between Aus1p, Pma1p, and ergosterol in non-budding or budding cells. WT_Aus1G/Pma1R cells were stained with filipin and then observed by focusing on the middle of the cells using a confocal fluorescence microscope. In non-budding or budding cells, Person’s correlation coefficient (PCC) between the fluorescent signals obtained with Aus1p-GFP and Pma1p-mCherry were indicated on the right side of the image. **(E)** Magnified image of each area enclosed by the dashed lines in **(D)**. Sterol was colored red or blue to facilitate comparison to the fluorescence of GFP (green) and mCherry (magenta). Intensity profiling of each fluorescence pattern was carried out along the dashed arrows and indicated in the corresponding light panel, respectively. **(F)** Scatterplots of green and cyan, or magenta and cyan pixel intensities in budding cells of **(D)**. PCC between Aus1p-GFP and Filipin, or Pma1p-mCherry and Filipin were graphically showed. All scale bars represent 2.5 µm.

Because the association of Pma1p with DRMs was not affected by *ERG25* knockdown (Figure 3A), we investigated whether *ERG25* knockdown influenced the localization of Pma1p to the plasma membrane. We constructed a Tet-ERG25 strain that co-expressed Aus1p-GFP and Pma1p-mCherry by inserting sequences encoding the indicated fluorescent tag in-frame and downstream of the respective genes. The resulting strain encoded C-terminally tagged fusion proteins from the endogenous loci. We then observed cells of this strain using fluorescence microscopy to assess the intracellular localization of Pma1p. The fluorescence of Pma1p-mCherry was detected on the plasma membrane and in the vacuole in the absence of Dox. In the presence of Dox, the fluorescence of Aus1p-GFP on the cell surface disappeared, while that of Pma1p was maintained on the cell surface, although the fluorescence signal detected in the vacuole increased (Figure 4A). These results indicate that the localization of Pma1p to the plasma membrane was largely unaffected by *ERG25* knockdown.

Most plasma membrane proteins are compartmentalized in distinct domains on the plasma membrane, and their distribution is influenced by the lipid composition of the plasma membrane (Spira et al., 2012). Therefore, we speculated that Aus1p and Pma1p could be normally located in distinct domains of the plasma membrane, that have distinct lipid compositions, and that ergosterol is an important element in the distribution of Aus1p-associated domains. To confirm this hypothesis, we compared the distribution pattern of Aus1p and Pma1p on the plasma membrane by real-time observation of cells co-expressing *AUS1-GFP* and *PMA1-mCherry*, using high-resolution confocal microscopy. The fluorescence of Aus1p-GFP and Pma1p-mCherry were detected in the different region on the plasma membrane (Figure 4B). Further high-resolution observation clearly showed the difference in the distribution of Aus1p and Pma1p. In non-budding cells, the fluorescence of Aus1p-GFP exhibited a network-like localization pattern similar to that seen for Pma1p. Notably, however, most of the Aus1p-GFP fluorescence did not overlap with that of Pma1p-mCherry in the images that focused on the top and middle of the cell (Figure 4C). The fluorescence intensity profile and scatterplot of green (Aus1p-GFP) and magenta (Pma1p-mCherry) pixel intensities also clearly indicated a difference in the localization patterns of the two proteins. When daughter cells were smaller than mother cells, the fluorescence intensity of Aus1p-GFP was higher at the cell surface of daughter cells than in that of the mother cells, whereas that of Pma1p-mCherry was higher at the cell surface of mother cells (Figure 4D). These results indicated that Aus1p and Pma1p are compartmentalized to distinct domains on the plasma membrane under normal circumstances. Furthermore, to determine whether the differences in the localization patterns of Aus1p and Pma1p correlated with sterol distribution, we observed sterol by staining with filipin. In budding cells, filipin stained the cell surface of daughter cells more strongly than that of mother cells, and the filipin staining pattern was similar to the fluorescence pattern of Aus1p (Figure 4D, bottom panel). Furthermore, in the magnified image of budding cells, the fluorescence intensity profile indicated that the fluorescence of Pma1p-mCherry did not overlap with the distribution of sterol, while that of Aus1p-GFP showed partial overlap with the filipin-stained region (Figure 4E). To quantify the extent to which Aus1p and Pma1p are in close proximity to sterol-rich regions on the plasma membrane, we calculated Pearson’s correlation coefficients (PCC) between fluorescene of Aus1p-GFP and Filipin, or that of Pma1p-mCherry and Filipin. The value of PCC between Aus1p-GFP and Filipin was higher than that of PCC between Pma1p-mCherry and Filipin (Figure 4F). These results suggested that the Aus1p-containing domains are enriched with sterol compared to the Pma1p-containing domains.

**FIGURE 5:**
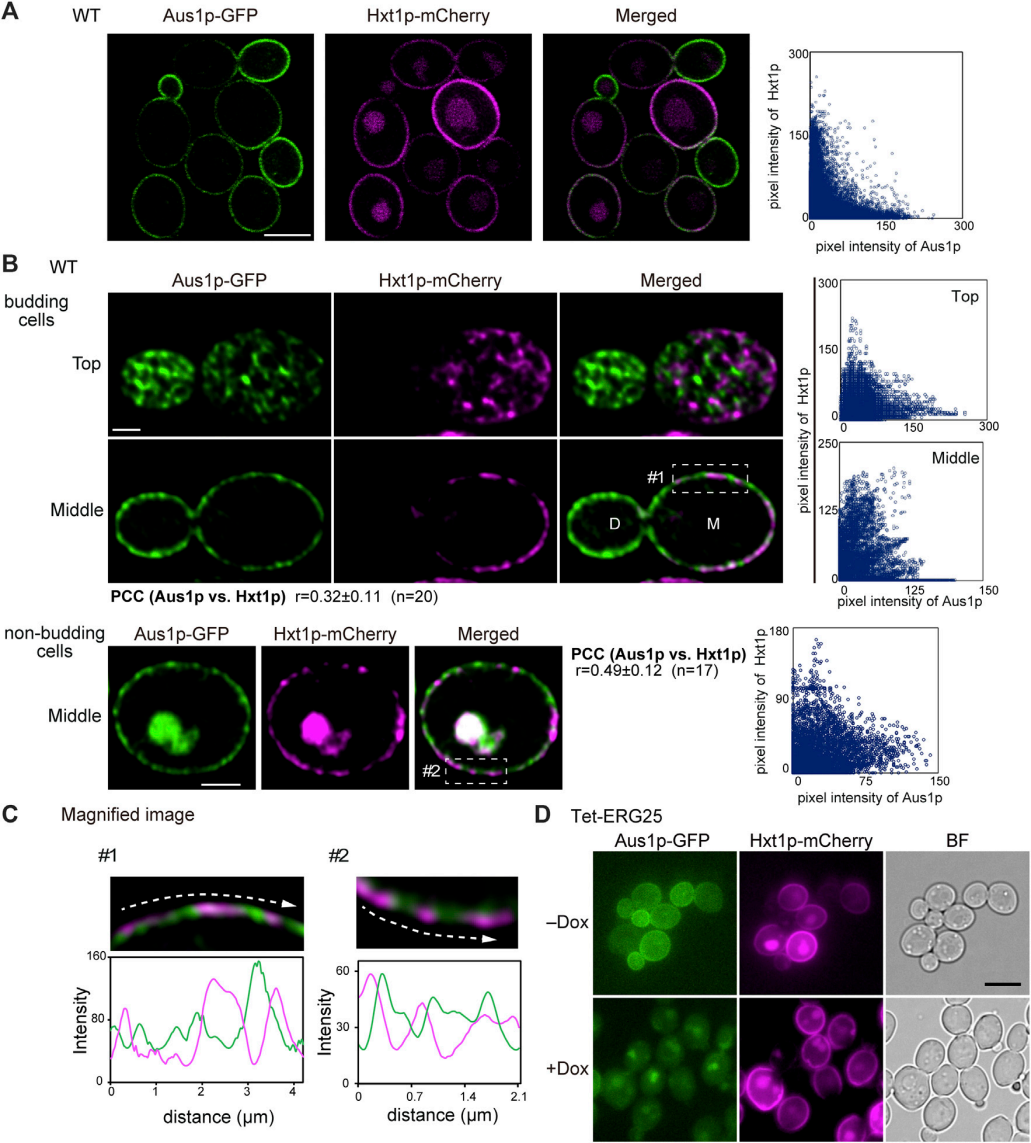
The plasma membrane distributions of Aus1p and Hxt1p are different, and *ERG25* knockdown has no effect on the localization of Hxt1p. **(A)** Distribution of Aus1p and Hxt1p on the plasma membrane. Wild-type cells co-expressing Aus1p-GFP and Hxt1p-mCherry (WT_Aus1G/Hxt1R) cells were grown to exponential phase at 30°C in minimal medium containing bovine serum. Scatterplot of green and magenta pixel intensities of Aus1p-GFP and Hxt1p-mCherry was shown in the right panel. Scale bar represents 2.5 µm. **(B)** WT_Aus1G/Hxt1R cells were observed at high resolution by focusing on the top and middle of the cells using a confocal fluorescence microscope. Scatterplots in each cell were represented in the right panel. PCC between the fluorescent signals obtained with Aus1p-GFP and Hxt1p-mCherry in budding or non-budding cells were indicated. Scale bars represent 1 µm. D, daughter cell; M, mother cell. **(C)** Magnified image of the area enclosed by the dashed lines in **(B)**. Intensity profiling of GFP (green) and mCherry (magenta) on the plasma membrane was carried out along the dashed arrows. **(D)** Effect of *ERG25* knockdown on the localization of Hxt1p. Tet-ERG25 cells that expresses Aus1p-GFP and Hxt1p-mCherry (Tet-ERG25_Aus1G/Hxt1R) were grown to exponential phase at 30°C in minimal medium containing 10% (v/v) bovine serum and observed using fluorescence microscopy after incubation with or without 20 μg/ml Dox for 3.5 h. Scale bar represents 2.5 µm.

To further confirm that *ERG25* knockdown does not influence protein targeting in sterol-poor regions of the plasma membrane, we investigated the localization of the hexose transporter Hxt1p, which (like Pma1p) has been reported to be localized preferentially on mother cells in *S*. *cerevisiae* (Malínská et al., 2003). Using confocal microscopy, detailed observation of wild-type cells endogenously co-expressing Aus1p-GFP and Hxt1p-mCherry revealed that the fluorescence of Hxt1p-mCherry was detected primarily on the mother cells, unlike the filipin staining pattern in Figure 4D and the distribution of Hxt1p-mCherry was distinct from that of Aus1p (Figure 5A). High-resolution observation also clarified the difference in the distribution between Aus1p and Hxt1p (Figure 5B). When focusing on the middle of the cell, the fluorescence of Hxt1p-mCherry was observed primarily in the region from which Aus1p-GFP was excluded (Figure 5C). This distinction is apparent in the corresponding fluorescence intensity profile, which showed that the peaks of Aus1p-GFP and Hxt1p-mCherry fluorescence do not align. These results suggested that Aus1p and Hxt1p are compartmentalized to distinct domains on the plasma membrane. We then investigated the effect of *ERG25* knockdown on the localization of Hxt1p. While the fluorescence of Aus1p-GFP on the cell surface disappeared in Tet-ERG25 cells grown in the presence of Dox, the fluorescence of Hxt1p-mCherry still was detected on the cell surface regardless of the presence of Dox (Figure 5D). Taken together, our results suggested that the effect of *ERG25* knockdown was specific to Aus1p-containing domains, and not to Pma1p- or Hxt1p-containing domains.

### MCC/Eisosome Structures Are Disrupted in *ERG25* Knockdown Cells

**FIGURE 6:**
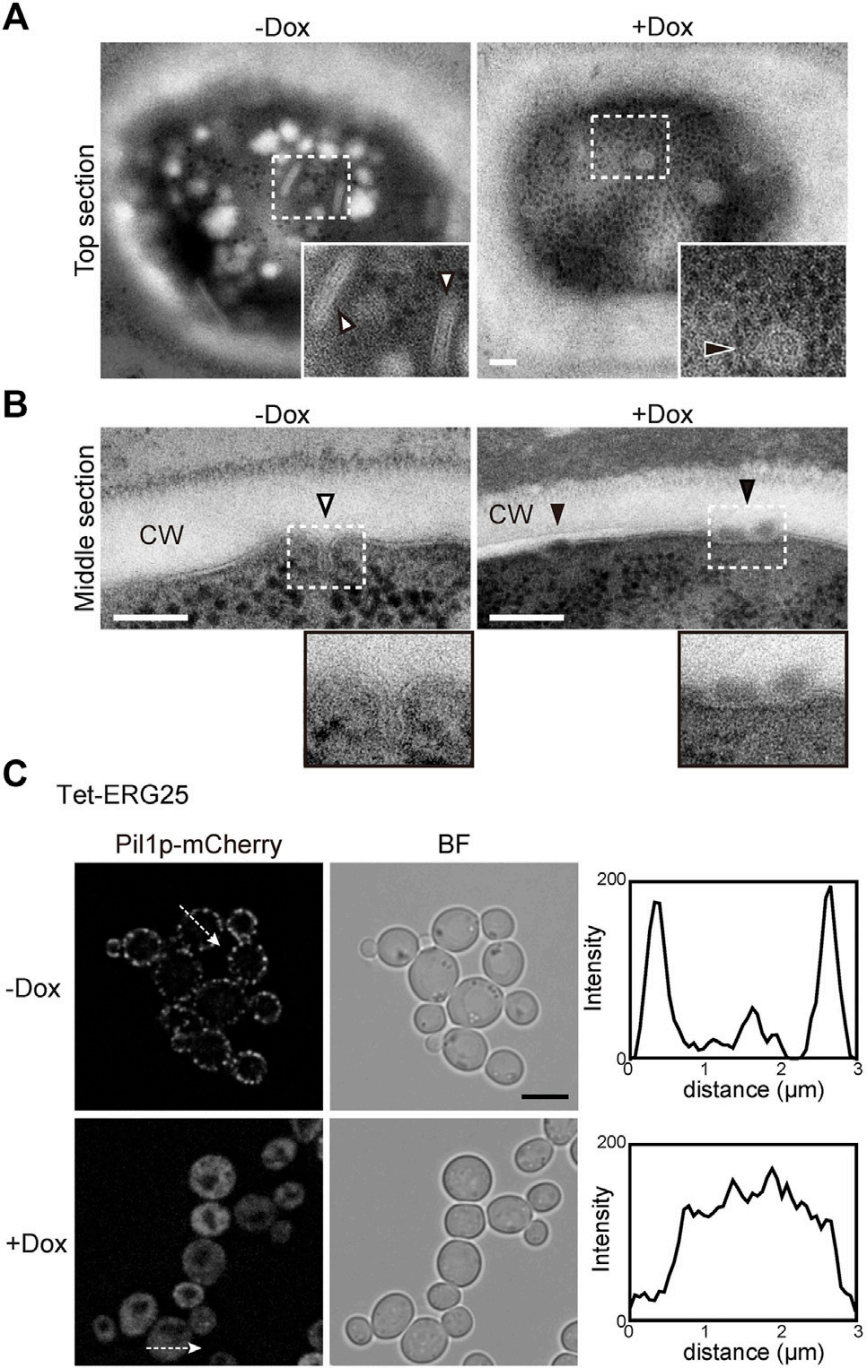
*ERG25* knockdown has effect on the structure of MCC/eisosomes. **(A)** Observation of furrow-like invaginations in Tet-ERG25 cells using transmission electron microscopy (TEM). Cells were incubated at 37°C in minimal medium containing 10% (v/v) bovine serum with or without Dox for 17 h, and then fixed. Ultrathin sections (70-nm thicknesses) of the cell surface were observed. The furrow-like invaginations in Dox-untreated cells are indicated by white arrowheads, and abnormal invaginations in Dox-treated cells are indicated by black arrowheads. The dashed lines surround areas shown at higher magnifications in the lower right. Scale bars represent 100 nm. CW; cell wall. **(B)** TEM images of the cells sectioned through the middle of the cells. **(C)** Effect of *ERG25* knockdown on the localization of Pil1p-mCherry. Tet-ERG25 cells expressing Pil1p-mCherry (Tet-ERG25_Aus1G/Pil1R) were grown to exponential phase at 37°C in minimal medium containing bovine serum and further incubated for 17 h in the absence or presence of Dox. Intensity profiling of Pil1p-mCherry was carried out along the dashed arrows. Scale bar represents 5 µm.

In *S. cerevisiae*, the MCC/eisosomes, which correspond to characteristic furrow-like invaginations in the plasma membrane (Strádalová et al., 2009), have been reported to be enriched in ergosterol (Grossmann and Malinsky, 2007). Therefore, to investigate whether *ERG25* knockdown affects the structure of the MCC/eisosomes, we observed the cell surface of Tet-ERG25 cells using transmission electron microscopy (TEM). In control cells untreated with Dox, furrow-like invaginations appeared as a bundle of lines with lengths of 130–200 nm, which are indicated by white arrowheads in tangential sections (Figure 6A); these invaginations exhibited depths of about 50 nm when viewed in transverse sections (Figure 6B). These structures were similar to the furrow-like invaginations corresponding to MCC/eisosomes of *S. cerevisiae* (Walther et al., 2006). By counting the number of invaginations in 13 *C. glabrata* cells, we quantified these structures at 3.2 ± 1.1/µm^2^ (mean ± standard deviation) in the absence of Dox and 0.3 ± 0.5/µm^2^ in the presence of Dox. In the presence of Dox, globular structures of 50–80 nm in diameter appeared on the cell surface, instead of furrow-like invaginations (black arrowheads in Figures 6A,B). The shape of these globular structures was similar to the shape of the remnants seen in *S. cerevisiae* with deletion of *PIL1*, which encodes a main organizer of MCC/eisosomes assembly (Moreira et al., 2012). These observations indicated that the influence of *ERG25* knockdown extended to the formation of furrow-like invaginations on the cell surface.

To further ascertain the effect of *ERG25* knockdown on MCC/eisosomes, we used fluorescence microscopy to observe the distribution of Pil1p in Tet-ERG25 cells. *PIL1* was endogenously tagged with sequences encoding mCherry so as to encode a C-terminally tagged protein. In wild-type cells, the fluorescence of Pil1-mCherry was detected in punctate compartments located on the cell surface in the presence or absence of Dox, suggesting that the localization of pil1p could not be affected by the addition of Dox (Supplementary Figure S5). On the other hand, in Tet-ERG25 cells, Pil1p-mCherry showed a punctate localization similar to wild-type cells (Figure 6C). In the absence of Dox, the fluorescence of Pil1p-mCherry was detected in punctate compartments located on the cell surface (Figure 6C). However, following *ERG25* knockdown with Dox, the punctate Pil1p fluorescence on the cell surface decreased, and fluorescence instead was observed in the cytoplasm. This result implied that *ERG25* knockdown causes diffusion of Pil1p from the plasma membrane to the cytoplasm.

### Aus1p Associates With Dynamic Domains That Occasionally Overlap With MCC/Eisosomes

**FIGURE 7:**
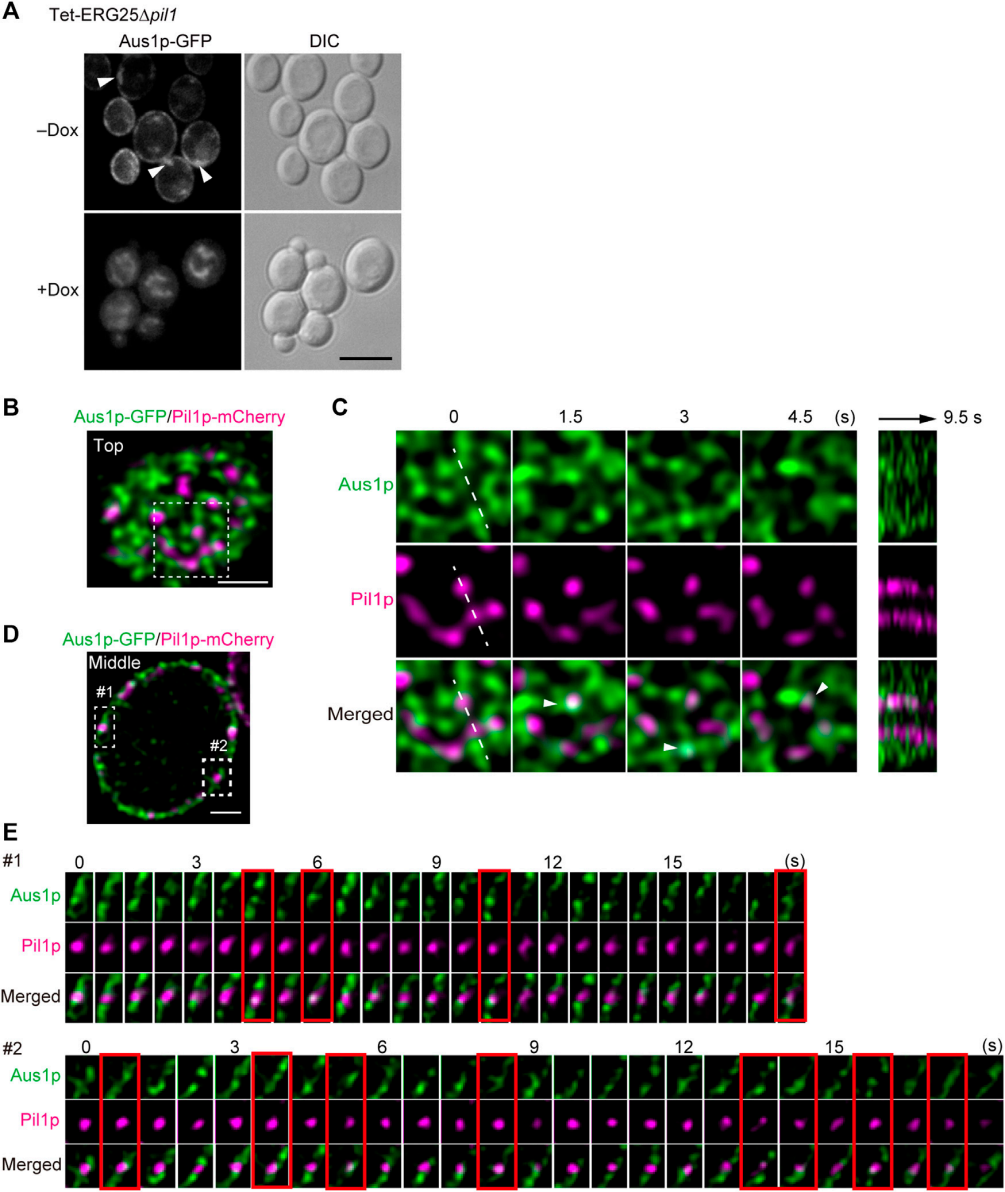
Aus1p is compartmentalized into plasma membrane domains distinct from MCC/eisosomes. **(A)** Effect of *PIL1* deletion on the localization of Aus1p. Tet-ERG25 *pil1*∆ cells expressing Aus1p-GFP (Tet-ERG25 *pil1*∆_Aus1G) were grown to exponential phase at 30°C in minimal medium containing bovine serum and observed using fluorescence microscopy after incubation with or without Dox for 3.5 h. Scale bar represents 5 µm. **(B)** Distribution of Aus1p and Pil1p on the plasma membrane. Wild-type cells co-expressing Aus1p-GFP and Pil1p-mCherry (WT_Aus1G/Pil1R) were incubated in the presence of serum and observed at the cell surface (Top) by high-resolution confocal microscopy. **(C)** Time-lapse imaging of the boxed region of **(A)**. Right panel shows the kymograph obtained by recording across the dashed line of the middle panels in a time-lapse image taken every 0.5 s. Co-localization of Aus1 and Pil1p appears in white, as indicated by arrowheads. **(D)** Distribution of Aus1p and Pil1p on the plasma membrane in the traverse region. **(E)** Time-lapse imaging of the boxed region of **(D)**. Images were taken at 0.75-s intervals. The images surrounded with red lines represent the co-localization of Aus1p-GFP and Pil1p-mCherry. All scale bars represent 2 µm.

Because the structure of MCC/eisosomes diffused in the cells in which *ERG25* was knocked down, the relationship between the diffusion and the mislocalization of Aus1p was investigated. We constructed Tet-ERG25∆*pil1*, a double-mutant strain (with downregulation of *ERG25* and knockout of *PIL1*) that expresses Aus1p-GFP, and compared the localization of Aus1p when this strain was grown with and without Dox. In the absence of Dox, Aus1p was observed to be localized on the cell surface, but fluorescent aggregates were observed as indicated by the white arrowhead (Figure 7A). When Tet-ERG25∆*pil1* was grown in the presence of Dox, Aus1p-GFP no longer localized to the plasma membrane, instead accumulating in the vacuole, as was seen previously in the case of *ERG25* knockdown. These observations show that the mislocalization of Aus1p by the knockdown of *ERG25* occurred independently of the aggregation of Aus1p on the plasma membrane, caused by the deletion of *PIL1*.

Given that the deletion of *PIL1* altered the inherent localization of Aus1p on the plasma membrane (as shown in Figure 7A), we investigated whether Aus1p associates with MCC/eisosomes on the plasma membrane. To compare the distributions of Aus1p and MCC/eisosomes, we performed dual-color imaging of Aus1p-GFP and Pil1-mCherry (a marker for MCC/eisosomes) in living cells. High-resolution imaging revealed that Aus1p fluorescence was detected primarily in the plasma membrane regions that did not contain Pil1p-mCherry (Figures 7B,D). Furthermore, we followed the behavior of Aus1p and Pil1p in detail by time-lapse observations. MCC/eisosomes (as indicated by Pil1p-mCherry) was static as reported in *S. cerevisiae* (Malínská et al., 2003), whereas Aus1p-GFP fluorescence migrated dynamically across the plasma membrane (Figure 7C) Supplemental Movie. Kymographs of the light panels also clearly highlight the dynamic differences between these domains, suggesting that Aus1p is dynamically localized to a domain that is distinct from MCC/eisosomes. However, the real-time imaging also showed some overlap between the fluorescence of Aus1p-GFP and that of Pil1-mCherry, as indicated by the white arrowheads in Figure 7C. Therefore, we performed time-lapse imaging of Aus1p-GFP, focusing on single MCC/eisosomes. We observed that some Aus1p-GFP was present in MCC/eisosomes for a short time, at intervals of about 1.5–4.5 s (frames surrounded by red lines in Figure 7E). These results suggested that Aus1p is mostly localized outside MCC/eisosomes, however, some Aus1p may access MCC/eisosomes occasionally.

## Discussion

Host-cholesterol uptake is one of the key survival strategies for successful infection, especially for *Candida glabrata*, which uses it to proliferate despite the presence of azole antifungal agents that inhibit ergosterol biosynthesis. In this study, among the screened ergosterol biosynthetic genes in *C. glabrata* (*ERG1, ERG7, ERG11, ERG25, ERG26*, and *ERG27*), we found that only the growth defects imposed by *ERG25* or *ERG26* knockdown were not rescued by the presence of serum (Figure 1B). Because the two genes are involved in a sequence of catalytic events, we concentrated our investigation on *ERG25*. In cells with little or no *ERG25* transcription, four observations were made: 4,4-dimethylzymosterol accumulates (Figure 3B); Aus1p-GFP delocalizes to the plasma membrane (Figure 2D); extracellular NBD-cholesterol is not uptaken (Figures 2A,B); the addition of serum cannot suppress their growth defects (Figure 1B). Based on these results, the effect of *ERG25* knockdown on Aus1p lipid domains was analyzed.

The selective interaction between ergosterol and sphingolipids has been reported to lead to phase separation into membrane domains with Liquid-ordered and Liquid-disordered like properties (Klose et al., 2010). The changes in sterol structure are known to destabilize or prevent the formation of liquid-ordered state domains (Xu et al., 2001; Megha et al., 2006). Since the liquid-ordered state conveys detergent resistance, we used DRMs to evaluate the liquid order state of membrane domains. In Tet-ERG25 cells under the Dox presence, Aus1p-GFP was scarcely detected in DRMs, whereas Pma1p had little or no effect on its distribution (Figure 3A). In addition, in Dox exposed Tet-ERG11 cells Aus1p-GFP was detected in the DRMs (Supplementary Data S6A). Furthermore, lipid analysis of DRMs showed that the abnormal sterol 4,4-dimethylzymosterol was detected as the major lipid component in Dox exposed Tet-ERG25 cells, whereas in Tet-ERG11 cells in the presence of Dox, the abnormal sterol, 4,14α-dimethylzymosterol was hardly or not detected in DRMs (Supplementary Data S6B). These results suggest that the loss of membrane localization of Aus1p-GFP was due to the presence of 4,4-dimethylzymosterol in the cell membrane, while in Tet-ERG11 cells in the presence of Dox, 4,14α-dimethylzymosterol hardly detected in the cell membrane, caused little effect in it and the localization of Aus1p-GFP was maintained.

**FIGURE 8:**
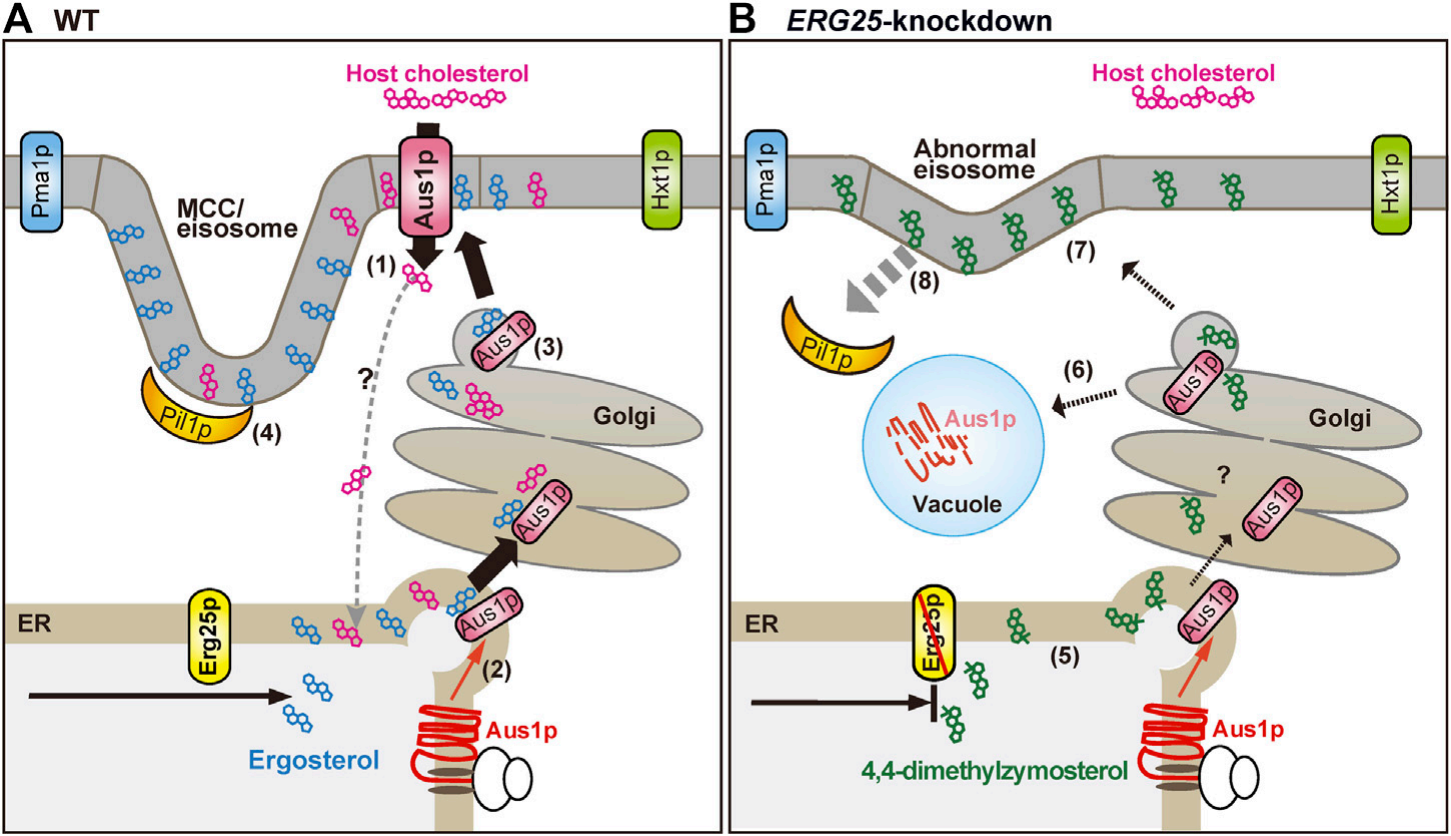
Schematic diagram showing the proposed effects of *ERG25* knockdown on host-cholesterol uptake. **(A)** Ergosterol and cholesterol interact with sphingolipid to form lipid domains (liquid-ordered state domain). Host-cholesterol is incorporated from serum via Aus1p, which is normally localized on the plasma membrane (1) and then transported to the endoplasmic reticulum (ER), although the transport route is unknown. In *S*. *cerevisiae*, the association of proteins with lipid domain occurs during trafficking from the ER to the plasma membrane *via* the Golgi. Therefore, newly synthesized Aus1p is inferred to be transported from the ER to the Golgi while being incorporated into lipid domains containing endogenous ergosterol (2, 3). Pil1p localizes to the membrane compartment of Can1p (MCC)/eisosomes that are enriched in ergosterol, generating furrow-like plasma membrane invaginations (4). Aus1p-associated domains are distinct from membrane compartment of Pma1p (MCP), membrane domains containing Hxt1p, and MCC/eisosomes. **(B)** In *ERG25* knockdown cells, 4,4-dimethylzymosterol is contained in lipid domains in place of ergosterol (5), but the domains are not allowed to fit the Aus1p due to 4,4-dimethylzymosterol. Therefore, newly synthesized Aus1p is unable to associate with lipid domain, and is missorted to the vacuole, resulting in the degradation (6). 4,4-Dimethylzymosterol, which is transported to the plasma membrane (7) and the release of Pil1p from the MCC/eisosomes (8). However, there is no effect on the localization of membrane proteins Pma1 and Hxt1.

The expression of Aus1p is regulated by Upc2A and Upc2B (Nagi et al., 2013), but the mechanism whereby Aus1p is transported to the plasma membrane remains unclear. The present study revealed that DRMs are required for the proper transport of Aus1p to the plasma membrane, as demonstrated by our analysis of cells knocked down for *ERG25* expression. We summarize our model for the transport of Aus1p in Figure 8. In *S. cerevisiae*, the association of membrane proteins with DRMs occurs in the ER or Golgi during intracellular transport (Bagnat et al., 2000; Okamoto et al., 2006), and some plasma membrane proteins are excluded from DRMs and are missorted to the vacuole from the late Golgi in the cells deleted for *ERG6,* which encodes an enzyme catalyzing a late step in ergosterol biosynthesis (Bagnat and Simons, 2002; Umebayashi and Nakano, 2003). Similar to ∆*erg6* cells, we showed, using *C. glabrata ERG25* knockdown cells, that Aus1p is mislocalized to the vacuole instead of the plasma membrane (Figure 2E). Therefore, in *ERG25-*knockdown cells, the association of Aus1p with DRMs in the ER and Golgi is prevented, and Aus1p appears to migrate from the late Golgi to the vacuole. Another experiment using *C. glabrata* supports this model. Specifically, while in the Tet-ERG25 cells absence of Dox, the deletion of *PIL1* affects the distribution of Aus1p in the plasma membrane after intracellular transport, the deletion of *PIL1* in combination with *ERG25* knockdown causes the mislocalization of Aus1p to the vacuole, similarly to the knockdown of *ERG25* alone (Figure 7A). These results suggest that Erg25p is a factor that transports newly synthesized Aus1p from the ER to the plasma membrane, and that Pil1p is not associated with this process. However Pil1p may be involved in the localization of Aus1p after it reaches the plasma membrane.

In *S*. *cerevisiae*, ergosterol has been reported to be required for the proper localization of the MCC-associated proteins Can1p and Sur7p, as demonstrated using strains deleted for non-essential ergosterol biosynthesis genes such as *ERG6* and *ERG24* (Malínská et al., 2003; Grossmann et al., 2008)*.* However, it is not yet clear whether ergosterol is required for the formation of eisosomes, because the deletion of these non-essential genes in *S*. *cerevisiae* did not result in an obvious defect in MCC/eisosomes formation or in the localization of Pil1p on the plasma membrane. *ERG25* knockdown in *C. glabrata* inhibited the normal formation of furrow-like invaginations (Figures 6A,B) and the retention of the MCC/eisosomes organizer Pil1p on the plasma membrane (Figure 6C), suggesting clearly the need for ergosterol in the formation and/or stability of eisosomes. The recruitment of Pil1p to the MCC/eisosomes is regulated by the phosphorylation of Pil1p via the Pkh1/2p kinases (Walther et al., 2007), which respond to the sphingolipid long-chain base (LCB) (Zhang et al., 2004). In *S*. *cerevisiae*, the *erg26* mutant cells, which are defective in the demethylation step of 4,4-dimethylzymosterol, exhibit a decrease in phytosphingosine-derived ceramide levels (Swain et al., 2002). Both ergosterol and cholesterol do not have a methyl group on the α-face, while 4,4-dimethyldysterol has a methyl group on the α-face (Supplementary Figure S7). Whether that clear structural difference interferes with the synthesis and association to sphingolipid is an important subject for further investigation.

Plasma membrane proteins are compartmentalized into distinct non-overlapping membrane domains. In addition to MCP and MCC/eisosomes, three non-overlapping plasma membrane domains have been identified in *S*. *cerevisiae*: membrane compartments containing the Target of Rapamycin kinase Complex 2 (TORC2; MCT) (Berchtold, 2009), the sterol transporters Ltc3/4 (MCL) (Murley et al., 2017), and the cell wall stress sensor Wsc1 (MCW) (Kock et al., 2016). These domains are characterized by their morphology and dynamics; the MCP shows a network-like distribution, while others show punctate distributions. Moreover, MCC/eisosomes, MCP, and MCL are static (Malínská et al., 2003; Murley et al., 2017), while the others are dynamic (Berchtold et al., 2012; Kock et al., 2016). Our observations indicate that the Aus1p-associated domain has a network-like distribution and is dynamic (Figures 4C, 7C). Therefore, we propose that the Aus1p-associated domain constitutes a novel lipid domain.

It is not clear what mechanism is used to form and maintain the heterogeneous distribution of membrane proteins. Notably, *ERG25* knockdown in *C. glabrata* influences the localization of Aus1p, but not that of Pma1p or Hxt1p (Figures 4A, 5D). These results support that ergosterol does not affect the localization of all membrane proteins. Additionally, *ERG26* knockdown also does not affect the localization of Hxt1p (Supplementary Figure S3B).

The Aus1p-related domain was shown to be preferentially concentrated in smaller daughter cells (Figure 4D). As reported about Pma1p in *S*. *cerevisiae*, the asymmetric localization of proteins in mother versus daughter cells is involved in promoting mother cell aging by affecting cellular homeostasis (Henderson et al., 2014; Yang et al., 2015), and sphingolipids contribute to maintain this asymmetric localization (Singh et al., 2017). On the other hand, ergosterol has been reported to be enriched in actively growing areas of the plasma membrane in various fungi, including *S*. *cerevisiae*, *C. albicans*, *Aspergillus nidulans*, and *Cryptococcus neoformans* (Bagnat and Simons, 2002; Martin and Konopka, 2004; Nichols et al., 2004; Pearson et al., 2004). Furthermore, many proteins identified to be enriched in daughter cells are needed for the emergence, construction, and division of the bud in *S*. *cerevisiae* (Yang et al., 2015). The Aus1p-associated domain is richer in ergosterol than the MCP in *C*. *glabrata* (Figures 4E,F). Therefore, the distribution of Aus1p in the cells appear to be correlated to the distribution of ergosterol. Although further studies are needed to clarify the mechanism by which Aus1p is distributed preferentially in daughter cells, we suggest that this Aus1p distribution bias to growing cells may allow cholesterol taken up from the host to be efficiently used for cell membrane synthesis in *C*. *glabrata*.

Recent studies also suggest that MCC/eisosomes act as reservoir domains for nutrient transporters, protecting them from endocytosis in response to nutrient starvation. The distribution of nutrient transporters to MCC/eisosomes has been suggested to be dependent on their conformational changes occurring upon substrate binding (Bianchi et al., 2018; Busto et al., 2018; Gournas et al., 2018; Moharir et al., 2018). For example, in *S*. *cerevisiae*, methionine permease Mup1p has been shown to be relocated from the MCC/eisosomes to a unique network-like domain at the plasma membrane in the presence of methionine (Busto et al., 2018). In recent years, MCC/eisosomes have been clarified to interact with MCT domains in the control of sphingolipid biosynthesis under conditions of membrane stress (Bartlett et al., 2015). Some MCT-associated proteins are spatially overlapped with MCC/eisosomes, and accumulate in a few large clusters reminiscent of eisosome remnants in ∆*pil1* cells (Bartlett et al., 2015). In our experiments with *C*. *glabrata*, some Aus1p was occasionally co-localized to MCC/eisosomes (Figure 7E) and accumulated in eisosome remnants-like structures in ∆*pil1* cells (arrowheads in Figure 7A). Therefore, Aus1p-associated domains may functionally be associated with MCC/eisosomes in *C*. *glabrata*. In *S*. *cerevisiae*, the transcription factors Upc2p and Sut1p, which regulate the expression of *AUS1* and of ergosterol biosynthesis genes in response to intracellular ergosterol abundance, regulate the expression of the genes encoding MCC/eisosome organizers, *NPC102* and *FHN2* (Wilcox et al., 2002; Foster et al., 2013), suggesting the involvement of the MCC/eisosomes in ergosterol homeostasis. We have observed that *C. glabrata* Aus1p localizes to novel network-like domains in the presence of serum. Although the specific role played by MCC/eisosomes to Aus1p localization requires further scrutiny, here, it is suggested that this role extends to sterol homeostasis mediated by the localization of Aus1p.

Clinical isolates of azole-resistant *C. glabrata* grow well in medium containing host serum even though they have lost the ability to synthesize endogenous ergosterol (Hanauer, 1992; Bard et al., 2005; Khan et al., 2014). Because such sterol-requiring strains are not able to grow in the medium commonly used for diagnostic examination (Hanauer, 1992; Khan et al., 2014), consequently, the sterol-requiring strains are likely to be overlooked in the culture examinations of patients with candidemia. Therefore, the real incidence of *C. glabrata* infection must be higher than what can be deduced from the published detection rate. The key players underlying cholesterol uptake, once identified and characterized, are thus likely to constitute promising new drug targets, especially for the drug resistant strains against to ergosterol associates. Similarity analyses suggested that the amino acid sequence of Erg25p is more highly conserved than Erg11p and Erg26p (for example, in *Aspergillus fumigatus*, *Coccidioides immitis* and *Cryptococcus neoformans*) and is less similar to its human orthologue (CNBC4830) (Supplementary Figure S8; Supplementary Table S2). Additionally, *ERG25* is the most highly conserved of the *ERG* genes examined among the fungi, and the least closely related to its human orthologue. Based upon these results, Erg25p has potential as a target for the development of new allosteric antifungal agents.

## Data Availability

The original contributions presented in the study are included in the article/Supplementary Material, further inquiries can be directed to the corresponding author.
