# Supplementary material for: Erg25 Controls Host-Cholesterol Uptake Mediated by Aus1p-Associated Sterol-Rich Membrane Domains in Candida glabrata
Source: Front Cell Dev Biol. 2022 Mar 24;10:820675. doi: 10.3389/fcell.2022.820675 (PMC8988197; doi:10.3389/fcell.2022.820675)
Supplement: Supplementary file 2 [file DataSheet1.docx]

Supplementary Figures

#

**Supplementary Figure 1.** (A) Schematic diagram of Tetracycline (tet)-regulated expression system. The Tet-off promoter was inserted into the promoter site of each *ERG* gene with *HIS3* as a transformation marker. Doxycycline (Dox) is a derivative of tetracycline. When Dox is absent, a tetracycline-controlled transactivator (tTA) binds to *tetO* (tetracycline operator) and *ERG* genes are transcribed. In the presence of Dox, Dox inhibits the binding of tTA to *tetO* and represses transcription of *ERG* genes. (B) The transcription of *ERG* gene is knock downed by addition of Dox in each Tet-ERG strains. Wild type (KUE100) or Tet-ERG strains were cultured in SD medium with or without Dox at 37°C for 4 h. Total RNA was extracted and *ERG* transcripts quantified by qRT-PCR relative to the *TEF1* internal control. Open and shaded bars represent the relative amount of mRNA in the absence and presence of Dox, respectively. In the condition without Dox (-Dox), mRNA level of the genes in each Tet-knock down strains was different from those in wild-type cells due to the regulation of gene expression by the Tet-off promoter. Error bars represent the SD from three independent experiments. The PCR conditions were as follows: pre-denaturation at 95°C for 1 min, followed by 40 cycles of denaturation at 95°C for 15 s and annealing/extension at 60°C for 1 min.

**Supplementary Figure 2.** Growth of the deletion strains of *ERG11* or *ERG25*. Wild-type cells (CBS138) were grown on the minimal medium plate (SD), ∆*erg11* cells on SD containing 10% serum (SD+serum), and ∆*erg25* cells on SD containing 50 µg/mL fluconazole (FCZ) and 10% serum (SD+FCZ+Serum) for 1 day. Cells were suspended in water at OD_600_ of 0.5 and 4-fold serial dilutions were spotted on SD, SD+Serum, SD+FCZ+Serum, or rich medium (YPD). Cells were incubated for 2 days at 37°C. The experiment was conducted three times.


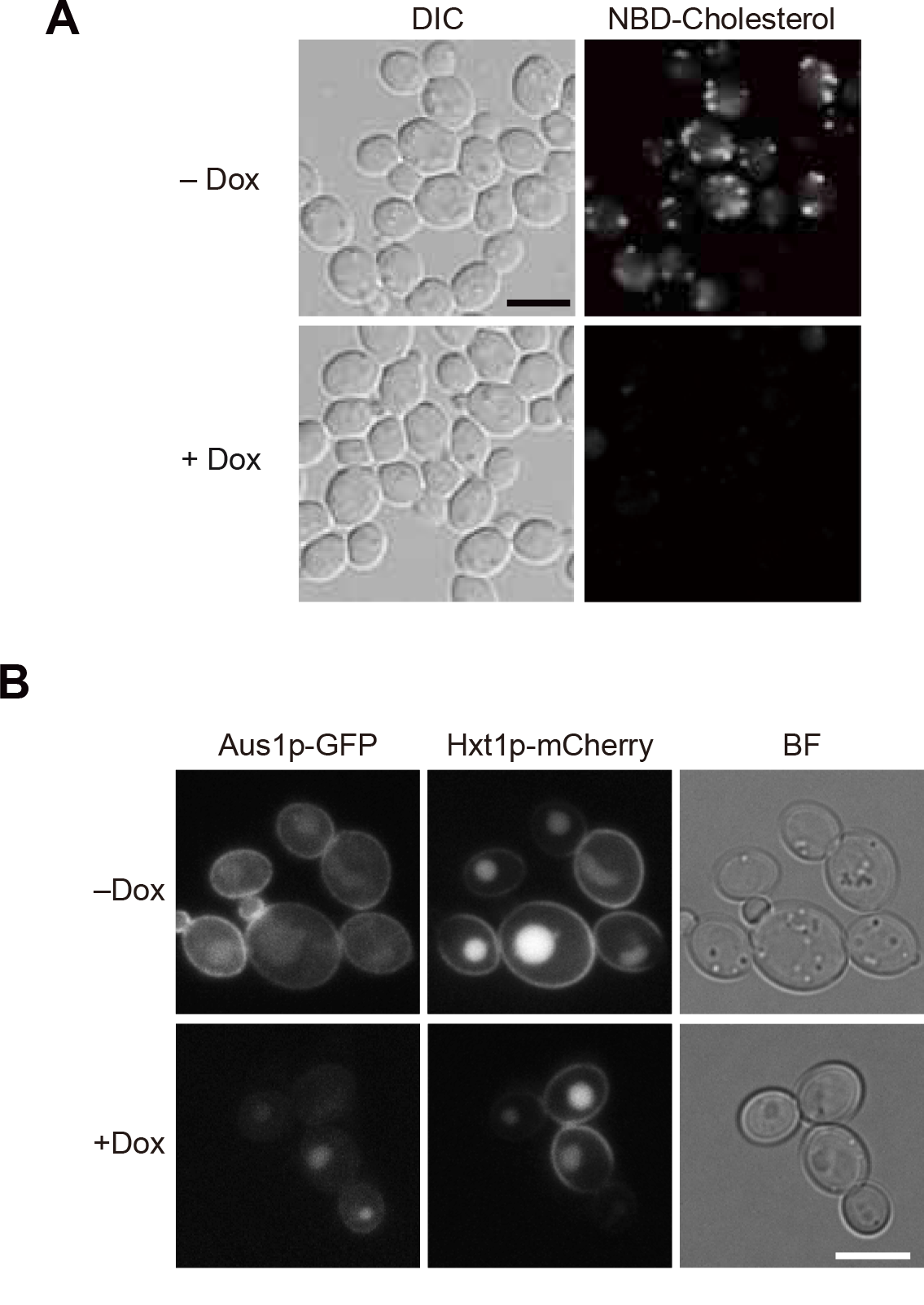


**Supplementary Figure 3.** *ERG26* knockdown causes defects in cholesterol uptake and the localization of Aus1p. **(A)** Tet-ERG26 cells were incubated with NBD-cholesterol in the presence or absence of Dox for 17 h in SD medium containing serum. NBD-cholesterol within the cells was observed under a fluorescence microscope. **(B)** Tet-ERG26 cells co-expressing Aus1p-GFP and Hxt1p-mCherry were incubated with or without Dox for 5.5 h in the presence of serum.

**Supplementary Figure 4.** The raw data of Figure 3A.

**Supplementary Figure 5.** The localization of Pil1p-mCherry in wild type cells in the presence of Dox. Wild type cells expressing Pil1p-mCherry (WT_Aus1G/Pil1R) were grown to exponential phase at 37 °C in minimal medium containing bovine serum and further incubated for 17 h in the absence or presence of Dox. Scale bar represents 5 µm.

**Supplementary Figure 6.** *ERG11* knockdown has no effect on the association of Aus1p with DRMs. **(A)** Tet-ERG11 cells were grown to the exponential phase at 37°C in SD containing serum, and then incubated with or without Dox for 5.5 hr. The cells were disrupted with glass beads, extracted with 1%TX-100, and subjected to Optiprep density gradient centrifugation. Seven fractions were collected and analyzed by Western blotting with antibodies against GFP and Pma1p. Fractions 2 contained DRMs. **(B)** Sterol analysis of whole cells and DRMs in Tet-*ERG11.* DRMs were isolated after the centrifugation by Optiprep density gradient and then analyzed by GC. 5-α-cholestane was used as internal standard.

**Supplementary Figure 7.** Chemical structures od ergosterol, cholesterol, and 4,4-dimethylzymosterol. Three dimensional structures are drawn with MolView. Allows show methyl groups at 4-position.

**Supplementary Figure 8.** Evolutionary relationship between taxa. The evolutionary history was inferred using the neighbor-joining method [1]. The optimal tree with a branch length sum of 2.89119262 is shown. The tree is drawn to scale, with branch lengths in the same units as those of the evolutionary distances used to infer the phylogenetic tree. The evolutionary distances were computed using the Poisson correction method [2] and are in the units of the number of amino acid substitutions per site. The analysis involved 10 amino acid sequences. All positions containing gaps and missing data were eliminated. There were a total of 313 positions in the final dataset. Evolutionary analyses were conducted using MEGA7 software [3]. Species are abbreviated as follows: A.f, *Aspergillus fumigatus*; C.a, *Candida albicans*; C.gl, *C. glabrata*; C.gu, *C. guilliemnon*dii; C.p, *C. parapsilos*is; C.t, *C. tropicalis*; Cr.n, *Cryptococcus neoformans*; S.c, *Saccharomyces cerevisiae*; and H.s, Homo sapiens.

[1] Saitou N, Nei M. (1987) The neighbor-joining method: A new method for reconstructing phylogenetic trees. Mol Biol Evolution 4:406-425.

[2] Zuckerkandl E, Pauling L. (1965) Evolutionary divergence and convergence in proteins. Evolving Genes and Proteins, eds Bryson V, Vogel HJ (Academic Press, New York), pp 97-166.

[3] Kumar S, Stecher G, Tamura K. (2016) MEGA7: Molecular Evolutionary Genetics Analysis version 7.0 for bigger datasets. Mol Biol Evolution 33(7):1870-4. doi: 10.1093/molbev/msw054.

**Supplementary Table 2.** BLAST search the genes of *C*. *glabrata* versus of other species.
